# Autoimmune HLA alleles and neoantigens predict myelodysplastic syndrome outcomes after allogeneic HSCT: A CIBMTR analysis

**DOI:** 10.1016/j.isci.2025.114326

**Published:** 2025-12-18

**Authors:** Timothy Sears, Razelle Kurzrock, Tao Zhang, Jing Dong, Stephen R. Spellman, Aaron M. Goodman, Yung-Tsi Bolon, Zhongyuan Chen, Paul Auer, Wael Saber, Hannah Carter

**Affiliations:** 1 Bioinformatics and Systems Biology Program, University of California, San Diego, La Jolla, CA, USA; 2 Department of Medicine, Medical College of Wisconsin Cancer Center and Genome Sciences and Precision Medicine Center, Milwaukee, WI, USA; 3 Linda T. and John A. Mellowes Center for Genomic Sciences and Precision Medicine, Medical College of Wisconsin, Milwaukee, WI, USA; 4 Division of Hematology and Oncology, Department of Medicine, Medical College of Wisconsin, Milwaukee, WI, USA; 5 CIBMTR® (Center for International Blood and Marrow Transplant Research), NMDP^SM^, Minneapolis, MN, USA; 6 Division of Blood and Marrow Transplantation, Department of Medicine, University of California, San Diego, La Jolla, CA, USA; 7 Cancer Center Biostatistics Shared Resource, Medical College of Wisconsin, Milwaukee, WI, USA; 8 Division of Biostatistics, Data Science Institute, Medical College of Wisconsin, Milwaukee, WI, USA; 9 CIBMTR® (Center for International Blood and Marrow Transplant Research), Medical College of Wisconsin, Milwaukee, WI, USA; 10 The Laboratory of Immunology, Moores Cancer Center and Department of Medicine, University of California, San Diego, La Jolla, CA, USA

**Keywords:** interventions, immunology, immune response

## Abstract

Allogeneic hematopoietic stem cell transplantation (allo-HSCT) offers curative potential for myelodysplastic syndrome (MDS), despite treatment-related mortality and relapse. We investigated how autoimmune human leukocyte antigen (HLA) alleles and mutanome-derived neoantigens influence post-transplantation outcomes. Donor and recipient HLA alleles, somatic mutations (508 genes; exome sequencing) and clinical covariates (*N* = 494 patients post-allo-HSCT [CIBMTR]) were evaluated. Class-I autoimmune alleles correlated with longer relapse-free survival (HR = 0.657, *p* = 0.011) (overall survival [OS]; HR = 0.787, *p* = 0.075). Improved calculated major histocompatibility complex-II (MHC-II) presentation of mutanome-derived neoantigens by donor HLA type correlated with longer OS (HR = 0.876, *p* = 0.034) (relapse-free survival; HR = 0.887, *p* = 0.083). Class-I auto-immune alleles plus chronic graft-versus-host disease (GVHD) enhanced the benefit of chronic GVHD alone for relapse-free survival (HR = 0.289, *p* < 0.001 vs. HR = 0.574, *p* = 0.031; comparison *p* = 0.021). Therefore, both autoimmune alleles and improved mutanome-derived neoantigen presentation correlated significantly and independently with relapse-free and OS, respectively, in a large multicenter group of MDS patients post-allo-HSCT. These factors warrant additional investigation for patient/donor selection.

## Highlights

• Class-I HLA autoimmune alleles correlate with longer RFS in MDS post-alloHSCT
• Improved donor MHC-II neoantigen presentation predicts longer survival in MDS post-alloHSCT
• Class-I autoimmune alleles enhance chronic GVHD’s graft-versus-leukemia effect in MDS

## Graphical abstract

**Figure:**
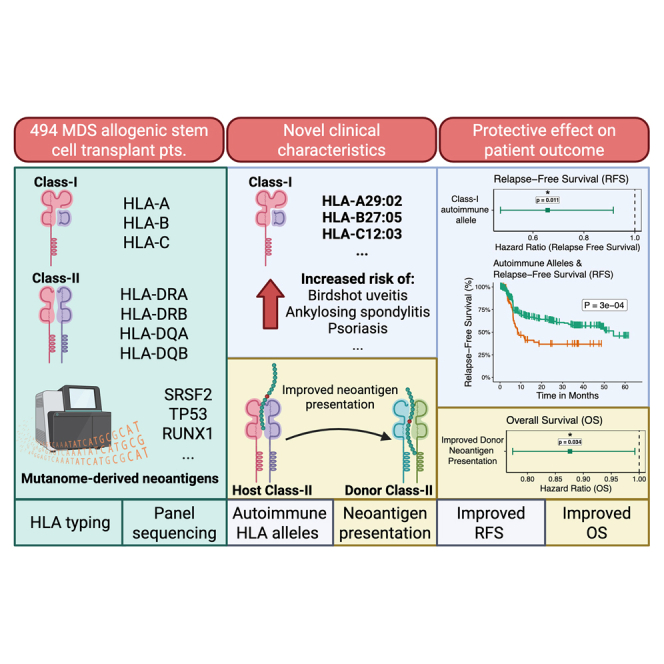

## Introduction

Allogeneic hematopoietic stem cell transplantation (allo-HSCT) has emerged as a potential curative therapy for eligible patients with high-risk myelodysplastic neoplasms (MDS). Patients receiving allo-HSCT have a prolonged overall survival (OS) but face serious potential side effects, including acute and chronic graft vs. host disease, and many patients still relapse.^1^

Many factors can influence the outcome of allo-HSCT for MDS, including disease stage, donor features, age, marrow blast counts, cytogenetics, International Prognostic Scoring System-revised (IPSS-R) category, matching of donor and recipient, time from MDS diagnosis to allo-HSCT,^2^ and specific types of mutations.^3^ However, these factors do not fully predict outcomes. Investigating additional predictive factors is therefore of potential significant value. Our previous pilot single-center study in 55 patients with acute myelogenous leukemia (AML) or MDS suggested that the presence of alleles associated with autoimmune diseases (autoimmune alleles) was correlated with better outcome. Additionally, we observed that the potential of the donor human leukocyte antigen (HLA) to better present neo-peptides representing driver mutations compared to the original host HLA was also correlated with better outcomes.^4^ Both of these factors are of interest for reasons grounded in the biology of relapse.

Strong associations between particular HLA alleles and the development of autoimmune disease have been observed for decades.^5^^,^^6^ This connection may derive from the presentation of self-peptides specific to certain HLA alleles.^7^^,^^8^ Notably, polygenic risk scores for autoimmune diseases may be higher in cancer exceptional responders^9^ though autoimmune disease may also increase cancer risk.^10^ Along these lines, the role of graft-versus-host disease in relapse and survival after allo-HSCT is important. Patients with acute graft-versus-host disease (aGVHD) grades 2–4 aGVHD are at a significantly higher risk of mortality.^11^^,^^12^^,^^13^ Similarly, the development of chronic graft-versus-host disease (cGVHD) results in both an increase in late non-relapse mortality and a decrease in quality of life.^14^^,^^15^^,^^16^ The development of cGVHD, however, may also increase relapse-free survival, presumably due to the graft-versus-leukemia (GvL) effect.^17^^,^^18^^,^^19^ GvL is mediated by restored T cell reactivity against malignant cells, which relies on antigen presentation via human leukocyte alleles (HLA).^20^ Prevention of graft rejection following allo-HSCT is also dependent on closely matching patient and donor HLA alleles, implicating transplanted donor HLA alleles in both anti-cancer immunity and auto-immunity.^21^^,^^22^

The ability of the major histocompatibility complex (MHC) to present specific cancer mutanome-derived neoantigens has also been implicated in the emergence of tumors or in immune escape after treatment in the case of poor antigen presentation.^23^^,^^24^ Specifically, a residue-centric patient MHC presentation score (termed the Patient Harmonic-mean Best Rank [PHBR] score) that describes a person’s ability to present specific cancer mutations to T cells given their patient-specific HLA alleles (with lower PHBR indicating more efficient presentation) has been developed for MHC class-I and class-II. These scores were found to correlate with the likelihood of mutations to emerge in a patient’s tumor.^23^^,^^25^ Poor presentation of driver mutation-derived neo-antigens by MHC may also explain why some tumors (even with a high tumor mutational burden) do not respond to immune checkpoint blockade.^26^

Herein, we hypothesize that the presence of autoimmune alleles as well as the capability to present mutanome-derived neo-antigens post allo-HSCT would independently correlate with improved relapse-free survival and OS, respectively, in a large muti-center cohort of patients with MDS.

## Results

We analyzed a cohort of 494 MDS patients receiving allo-HSCT with accompanying gene mutation data and HLA typing (Table 1). Of these patients, 306 (61.9%) had a detectable nonsynonymous mutation totaling 449 mutations, with some patients having up to five. Of these 449 nonsynonymous mutations across the cohort, 239 (53.2%) were missense, 194 (43.2%) were frameshift/nonsense mutations, and 16 (3.6%) were in-frame insertion/deletions. Most patients had 8/8 matching HLA alleles from their donor (91%), leaving only a subset (9%) of patients to evaluate for differences in antigen presentation between host and donor. The median age of the cohort was 62, while the median donor age was 35, and the cohort was 36% female. Overall, 227 patients (46.0%) experienced aGVHD grades 2–4 with a median time to onset of 1.12 months; grades 3–4 occurred in 89 (18.0%) with a median time to onset of 1.07 months; and cGVHD occurred in 232 (47.0%) of patients with a median time to onset of 6.48 months. Relapse occurred in 174 patients (35.25%) after a median of 10.35 months, and death occurred in 278 of patients (56.3%) after a median of 14.21 months. Times above are from the date of transplant.

**Table 1:** Patient characteristics

Characteristics	Patients
No. of patients	494
No. of centers	93
Patient age (year)—median (min-max)	66 (22–78)
Sex—no. (%)	
Male	315 (64)
Race/ethnicity—no. (%)	
Caucasian, non-Hispanic	494 (100)
KPS no. (%)	
0–90	251 (51)
HCT-CI no. (%)	
0–2	171 (35)
3+	315 (64)
Missing	8 (2)
Pre-transplant therapies (%)	
HMA alone	340 (69)
Chemo alone	15 (3)
HMA plus chemo	34 (7)
Neither	93 (19)
Missing	12 (2)
MDS IPSS-R score pre transplant—no. (%)^27^	
Very low	57 (12)
Low	123 (25)
Intermediate	160 (32)
High	74 (15)
Very high	22 (4)
Missing	58 (12)
Time from diagnosis to HCT (month)—median (range)	18 (2–263)
Donor type—no. (%)^28^	
HLA-identical sibling	65 (13)
Other related	32 (6)
Well-matched unrelated (8/8)	353 (71)
Partially matched unrelated (7/8)	39 (8)
Mis-matched unrelated (≤6/8) or unknown	5 (1)
Stem cell source—no. (%)^29^	
Bone marrow	59 (12)
Peripheral blood	435 (88)
Regimen intensity—no. (%)^30^	
Myeloablative	127 (26)
Reduced intensity	308 (62)
Non-myeloablative	44 (9)
Missing	15 (3)
Year of HCT—no. (%)	
2014	91 (18)
2015	156 (32)
2016	118 (24)
2017	122 (25)
2018	7 (1)
Median follow-up of survivors (months)—median (range)	34.5 (3.2–62.7)
Class I 1+ autoimmune allele	350/494 (70.9%)
Class II 1+ autoimmune allele	175/494 (35.4%)
Average number of panel mutations^a^ (sd)	1.204 (0.645)
Median time to graft versus host diseases (95% CI)^11^^,^^14^	
Acute grades 2–4	1.12 months (1.02–1.18)
Acute grades 3–4	1.07 months (0.95–1.22)
Chronic	6.48 months (5.82–7.34)

Abbreviations: HMA, hypomethylating agents; HCT, hematopoietic stem cell transplantation; IPSS-R, international prognostic scoring system; KPS, Karnofsky performance status.

a Including frameshift mutations.

### Autoimmune alleles are associated with improved relapse-free survival and OS

**Figure 1:**
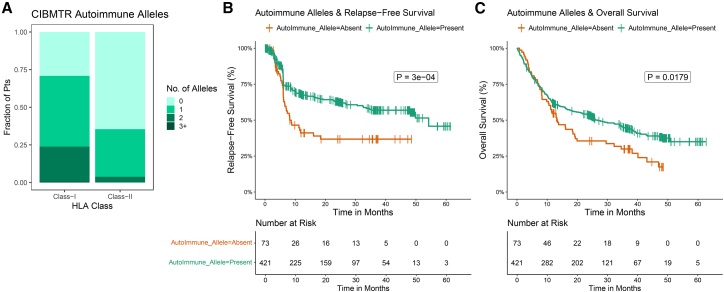
The presence of one or more autoimmune alleles is associated with improved relapse-free survival and OS in CIBMTR MDS patients (A) Frequency of autoimmune alleles by class in CIBMTR cohort. (B) Relapse-free survival stratified by presence or absence of any autoimmune allele. (C) OS stratified by presence or absence of autoimmune allele. *p* value calculated using log rank test.

Based on our auto-immune allele selection criteria (see “STAR Methods”), we identified 421 patients (85%) as having ≥1 autoimmune HLA allele, with 71% of patients harboring ≥1 class-I autoimmune allele, and 35% of patients with ≥1 class-II autoimmune allele (Table 2; Figures 1A, S1A, and S1B; and Table S1). The most common of these alleles was HLA-B07:02 with allele frequency (AF) of 0.141, followed by HLA-C05:01 (0.089) and HLA-C12:03 (0.0496), which are known to be associated with ankylosing spondylitis, multiple sclerosis, and psoriasis respectively. CIBMTR cohort autoimmune allele frequencies did not significantly differ from general population allele frequencies.^31^ We next compared our observed autoimmune allele frequencies to those in the acute myeloid leukemia (LAML) cohort within The Cancer Genome Atlas (TCGA); the incidence of both class-I and class-II autoimmune alleles were nearly identical in the TCGA LAML cohort (Figure S2).

**Table 2:** Autoimmune HLA allele overview

Allele	Primary risk association(s)	Cohort AF^a^	Population AF^b^
**Class-I Alleles**			

HLA-A29:02	birdshot uveitis^32^^,^^33^^,^^34^^,^^35^	0.0334	0.0353^31^
HLA-B27:05	ankylosing spondylitis^36^^,^^37^^,^^38^^,^^39^	0.0364	0.0373
HLA-B35:01	autoimmune hepatitis,^40^ Subacute thyroiditis^41^^,^^42^^,^^43^	0.0455	0.056
HLA-B^a^50:01	chronic spontaneous urticaria,^44^ myasthenia gravis^45^	0.0121	0.0105
HLA-B^a^51:01	Behçet’s syndrome^46^^,^^47^	0.0455	0.0473
HLA-B^a^13:02	psoriasis^48^^,^^49^^,^^50^	0.0253	0.0239
HLA-B^a^07:02	ankylosing spondylitis^51^^,^^52^	0.141	0.1306
HLA-C^a^12:03	psoriasis^53^^,^^54^^,^^55^^,^^56^	0.0496	0.0486
HLA-C^a^05:01	multiple sclerosis^5^^,^^57^	0.0891	0.0939

**Class-II Alleles**			

HLA-DRB1^a^08:01	primary biliary cirrhosis^58^	0.0172	0.0232
HLA-DRB1^a^13:02	autoimmune hepatitis type 1,^59^ dermatomyositis^60^	0.0395	0.0488
HLA-DRB1^a^08:03	multiple sclerosis,^61^ sarcoidosis^62^^,^^63^	0.00304	0.0024
HLA-DRB1^a^03:02	autoimmune hepatitis type 1^64^	0.00101	0.0003
HLA-DRB1^a^14:01	pemphigus vulgaris^65^^,^^66^	0.00202	0.0261

a Cohort AF refers to the within CIBMTR discovery cohort allele fraction of a given HLA allele.

b Population AF refers to the general population allele frequency reported in the Allele Frequency Net Database^31^ for individuals of European ancestry.

In our cohort of MDS patients, we found a statistically significant association between the presence of autoimmune alleles and longer relapse-free survival (HR = 0.5242, *p* < 0.001; Figure 1B) in a univariate Kaplan-Meier analysis. A significant correlation was observed between autoimmune alleles and longer OS as well (HR = 0.696, *p* = 0.0179; Figure 1C).

### Autoimmune alleles and other clinical variables are not associated with somatic mutations or quality of antigen presentation

Our cohort displayed a diverse mutational landscape. The top mutations—SRSF2, TP53, and RUNX1—were only present in 7%, 5%, and 4% of cases, respectively (Figure S3). This is consistent with existing investigations into MDS which have found low tumor mutational burdens spread across >30 key driver genes.^67^^,^^68^ The 38.1% of patients in our cohort with no nonsynonymous mutations were likely driven by mutations outside of our sequencing panel (see “STAR Methods”) or driven by copy number and epigenetic changes characteristic of MDS.^69^

We next measured the landscape of changes in antigen presentation caused by mismatched donor HLA alleles using the PHBR score (see “STAR Methods”).^23^^,^^25^ Briefly, PHBR scores were generated by taking the harmonic mean of computationally generated estimates of neoantigen binding affinity to a set of HLA alleles. Patients with one or more mismatched donor HLA alleles necessarily had a change in mutation presentation, where lower PHBR scores indicate better presentation (see “STAR Methods”). Changes in antigen presentation that move a given mutation into the lower gray quadrant (Figures S4A and S4B) reflect better presentation by donor HLA alleles. Overall, 16 (3.2%) patients had an improved donor allele PHBR-I score, 11 (2.2%) patients had a worse donor PHBR-I score, 9 (1.8%) patients had an improved donor allele PHBR-II score, and 8 (1.6%) patients had a worse donor PHBR-II score, when compared to baseline presentation of neoantigens with host HLA alleles. There was no association between specific mutations or overall mutational burden with any kind of GVHD or the presence/absence of autoimmune alleles (Figure S5). We found no association between changes in antigen presentability and the presence of autoimmune alleles, or with the development of acute or chronic graft versus host disease (Figure S6).

### Multivariable time-dependent cox proportional hazards analysis reveals class-I autoimmune alleles as independently protective against relapse

**Figure 2:**
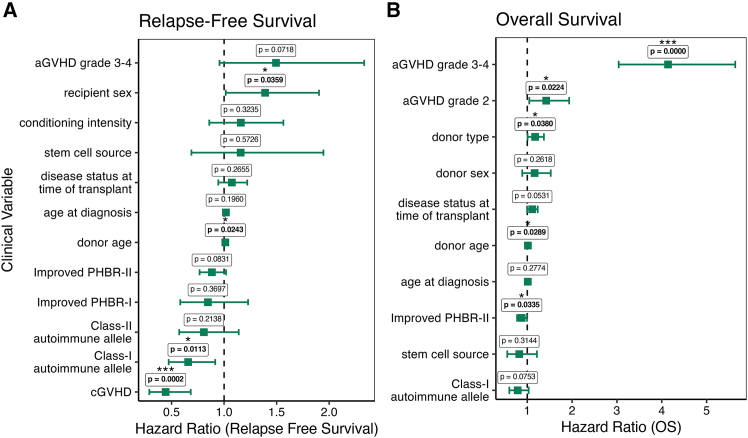
Backwards selection of clinical features in time-dependent multivariable CoxPH model reveals associations between novel clinical covariates and outcome. (A) relapse-free survival and (B) Overall survival. *p* values generated by likelihood ratio test, ∗ <0.05, ∗∗ <0.01, ∗∗∗ <0.001.

Class-I autoimmune allele presence was associated with longer relapse-free time (HR = 0.657, *p* = 0.011; Figure 2A) and trended toward significance for OS (HR = 0.787, *p* = 0.075). Consistent with earlier reports, cGVHD was strongly associated with longer relapse-free time (HR = 0.445, *p* < 0.001) but did not reach significance regarding OS, likely due to the opposing influence of GvL and graft-versus-host effects. Increased donor age was hazardous in both relapse-free (HR = 1.012, *p* = 0.024) and OS settings (HR = 1.013, *p* = 0.029). Glucksberg grades 2–4 and grades 3–4 aGVHD were both associated with a reduction of OS (HR = 1.420, *p* = 0.024; HR = 4.137, *p* < 0.001), but only grades 3–4 aGVHD trended toward an association with a reduction in relapse-free survival (HR = 1.492, *p* = 0.0718). Presence of class-I autoimmune allele was protective against relapse but not treatment-related mortality (TRM) (Figures S7A and S7B; *p* = 0.0142 and not significant, respectively, as was cGVHD, *p* = 0.0002 and not significant, respectively). We confirmed the impact of class-I autoimmune alleles and cGVHD on relapse only by performing a competing risk analysis^70^ with TRM and censored patients. Within this analysis, only in relapse were class-I autoimmune alleles and cGVHD protective (Figures S8A and S8B; *p* < 0.001, *p* < 0.001, respectively).

### Multivariable time-dependent Cox proportional hazards analysis reveals that change in neo-antigen presentation as reflected by PHBR-II scores independently associated with longer OS

We combined changes in antigen presentation and autoimmune alleles (now split by HLA class) with several clinical variables in a multivariable Cox model to determine if they are predictive of patient survival or relapse. Using a time-dependent backwards selection approach (“STAR Methods”), we found that improved PHBR-II presentation (a continuous score where positive values indicate enhanced MHC-II-based antigen presentation; see “STAR Methods”) was associated with longer OS (HR = 0.876, *p* = 0.034; Figure 2B), while improved PHBR-II presentation trended toward significance in relapse-free survival (HR = 0.887, *p* = 0.083). Due to the immunological nature of the significantly protective features (cGVHD, class-I autoimmune alleles, improved PHBR-II), we performed a similar time-dependent multivariable analysis with the manual inclusion of binary variables encoding post-treatment prophylactic GVHD immunosuppression (via PTCy) and pre-treatment ATG/Campath immunosuppression. We observed no significant changes to our findings (Figures S9A and S9B). Finally, we report no significant confounding clinical variables in patients with or without changes to their PHBR scores (Table S2).

### PHBR-based measures of overall immunogenicity were not uniformly associated with OS and relapse-free survival

None of our PHBR-based measures of overall immunogenicity, including the best presented neoantigen overall by donor, total sum of PHBR scores observed for all mutations for each patient, and the average of PHBR scores observed for all mutations for each patient, were predictive of relapse-free survival or OS in an initial univariate analysis (Figures S10A and S10B). This lack of predictive ability of the PHBR-based measures may be due to the limited panel of common driver mutations used in our study, which does not reflect the complete immunogenicity of a patient’s mutanome. Only when mismatched donor HLA alleles improved presentation over recipient HLA alleles did we see a protective effect. Due to this constraint, we repeated this analysis in an independent validation cohort consisting of 59 patients from the UC San Diego (UCSD) PREDICT trial (NCT02478931), using similar covariates (“STAR Methods”). We observed a non-significant protective effect of improved PHBR-II in the UCSD cohort for relapse-free survival and OS (Figures S11A and S11B), yet pooled meta-analysis (total improved PHBR-II *N* = 64; see “STAR Methods”) between the CIBMTR and UCSD cohorts demonstrated a significant association with both OS and relapse-free survival (Figure S11C; *p* < 0.05 and, *p* < 0.05, respectively).

### Pairwise interaction analysis between clinical variables supports a protective interaction between Class-I autoimmune alleles and cGVHD

**Figure 3:**
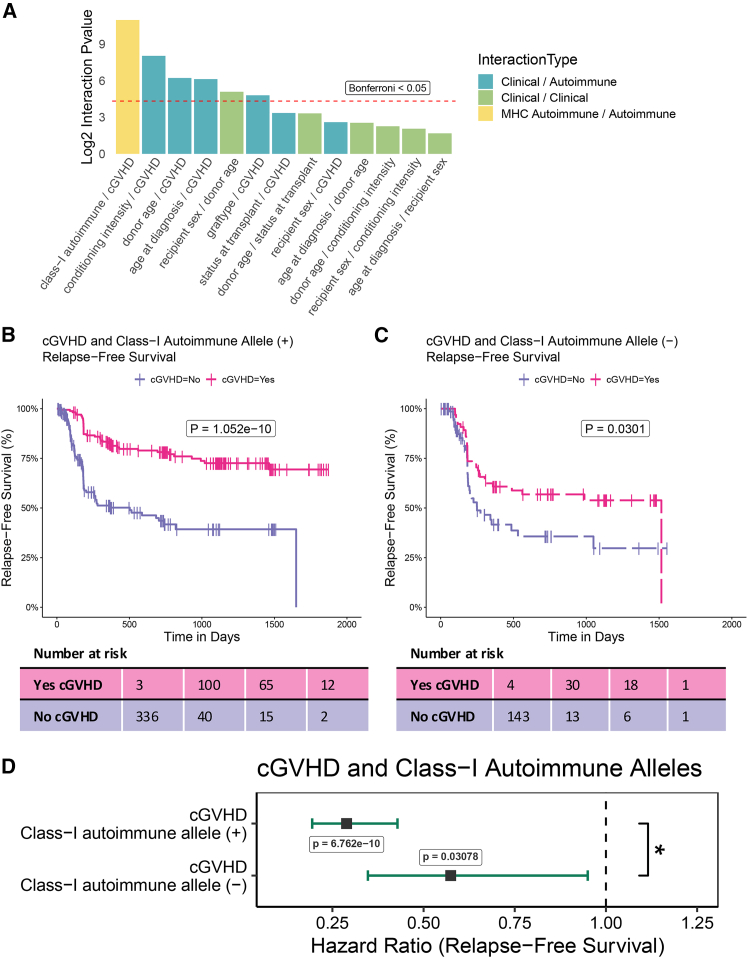
Interaction analysis of clinical features indicates class-I autoimmune alleles and cGVHD as key interactors in relapse-free survival (A) Pairwise interaction analysis of all clinical features passing backwards selection in time-dependent relapse-free survival analysis. *p* values are adjusted via Bonferroni correction. (B) Simon-Makuch plot of relapse-free survival of class-I autoimmune allele (+) CIBTMR patients stratified by cGVHD. *p* value computed by the Mantel-Byar test. Note that the Mantel-Byar method accounts for the time-dependent nature of cGVHD positive patients by counting them as “at-risk” only once the time of onset of their cGVHD has been met. (C) Simon-Makuch plot of relapse-free survival of class-I autoimmune allele (−) CIBTMR patients stratified by cGVHD. *p* value computed by the Mantel-Byar test. (D) Time dependent Cox proportional hazard of cGVHD with and without class-I autoimmune allele for relapse-free survival. *p* value computed by two-tailed Z-test, ∗ <0.05.

We next developed a putative mechanism for the protective nature of the presence of class-I autoimmune alleles against death from relapse. To accomplish this in an unbiased manner, each pair of clinical variables passing backwards time-dependent selection in the relapse-free survival setting was evaluated for potential interactions (“STAR Methods”). After multiple testing correction, we observed that class-I autoimmune alleles and cGVHD had the most significant interaction effect (Figure 3A). Due to the immortal time bias inherent in analyzing cGVHD, care had to be taken to understand how class-I alleles could be influencing the graft versus leukemia effect of cGVHD (“STAR Methods”). Briefly, all Cox models contained time-dependent covariate information, and Kaplan-Meier curves were substituted for Simon-Makuch^71^ plots where the accompanying log rank test was replaced by the Mantel-Byar^72^ test—both of which are more suitable for comparisons involving time-dependent covariates.

The impact of the presence of class-I autoimmune alleles on the mechanism of cGVHD was evaluated by splitting our cohort into class-I autoimmune present and absent strata. Once again, cGVHD was associated with significant improvement in relapse-free survival in both autoimmune allele groups (class-I autoimmune present, *p* < 0.0001; class-I autoimmune allele absent, *p* = 0.0301; Figures 3B and 3C). Interestingly, patients with a class-I autoimmune allele were significantly more protected by the development of cGVHD than those without (*p* = 0.021; Figure 3D). These data suggest that while class-I autoimmune alleles are not required to benefit from the anti-cancer effect of cGVHD, they robustly enhance the graft versus leukemia effect—possibly via impaired regulatory responses.^73^ This analysis was repeated using standard Kaplan-Meier curves with a 9-month landmark where the combination of class-I autoimmune alleles and cGVHD significantly outperformed either clinical characteristic alone (Figure S12A; *p* < 0.0001). To account for the possibility of an outlier driven effect, the analysis was repeated but with a bootstrapped subset (bootstrap *n* = 1,000) of the CIBMTR cohort where similar effects were observed (Figure S12B; *p* < 0.0001). We next confirmed the relapse-specific protective nature of this interaction by observing a significant protective effect of the combination of class-I autoimmune allele and cGVHD in relapse only, with no effect on TRM or incidence of censorship. This protective effect was significantly larger than its constituent features (Figures S13A–S13D). Finally, we replicated this finding in the smaller UCSD validation cohort where again, the combination of class-I autoimmune alleles and cGVHD was significantly associated with improved relapse-free survival when compared to the absence of either, both without bootstrapping (Figure S14A; *p* < 0.011) and with bootstrapping (*n* = 250) (Figure S14B; *p* < 0.0013). Hence, it appears that the combination of cGVHD and the presence of autoimmune alleles is highly predictive of a longer relapse-free survival.

## Discussion

MDS are a group of hematopoietic disorders characterized by ineffective blood cell production and a high risk of progression to AML. The standard of care for MDS includes high-dose chemotherapy to eradicate the patient’s diseased bone marrow, followed by allogeneic stem cell transplantation (allo-HSCT) from an HLA compatible donor. Despite its curative potential, allo-HSCT is complicated by TRM and does not eliminate the risk of relapse-related mortality (RRM). Advancements in conditioning regimens, supportive care, and post-transplant monitoring have significantly reduced TRM. However, RRM remains a significant challenge, with relapse of the underlying disease being the primary cause of treatment failure.

GVHD can develop following allo-HSCT and manifests in acute and chronic forms. While associated with TRM, there is an increasing appreciation for the anti-cancer effect of chronic cGVHD, a phenomenon termed the GvL effect. GvL involves a delicate balance between beneficial anti-leukemic effects and the detrimental consequences of cGVHD, where donor immune cells attack the recipient’s healthy tissues. cGVHD has been associated with a reduced risk of relapse, suggesting overlapping immune mechanisms between GVHD and GvL. While the GvL phenomenon has immense promise to reduce RRM, its mechanism of action is not yet fully understood. We speculate that a potential mechanism for the observed interaction between autoimmune-predisposing HLA alleles and cGVHD could involve alterations in regulatory immune responses.^6^ One hypothesis is that T cells in the context of these specific HLA alleles might exhibit reduced differentiation into regulatory T cells (Tregs), which are crucial for maintaining immune tolerance.^7^ Alternatively, given that class-I HLA molecules present antigens to CD8^+^ T cells,^74^ these autoimmune-associated alleles may present a broader repertoire of self or neoantigens, thereby enhancing CD8^+^ T cell alloreactivity and cytotoxic responses against residual leukemic cells. Such a scenario may, in turn, enhance the RRM reduction granted by cGVHD by potentially altering the GvL effect. These mechanisms are not mutually exclusive and may act in concert to modulate the GvL effect.

Our study revealed a previously unrecognized interaction between class-I HLA alleles associated with autoimmune diseases and the effectiveness of the GvL phenomenon. Patients with these specific HLA alleles demonstrated a significantly longer duration of relapse-free survival. These observations have significant implications for allo-SCT. Traditionally, HLA matching for transplantation prioritizes minimizing mismatches to reduce the risk of GVHD and other complications. However, our findings suggest that certain HLA mismatches, specifically those involving alleles associated with autoimmune diseases, might be beneficial in enhancing the GvL effect. This insight could lead to a shift in HLA matching strategies, where the presence of autoimmune-associated alleles might be considered favorable in certain contexts. While these results should be interpreted with caution due to the lack of inclusion of the critical covariate IPSS-M, they strongly highlight the need for further research to elucidate the mechanisms through which these HLA alleles influence the immune response post-transplantation. Furthermore, our findings highlight the need for further research to elucidate the mechanisms through which these HLA alleles influence the immune response post-transplantation. Understanding these pathways could pave the way for targeted interventions to harness the beneficial aspects of the GvL effect while mitigating the risks of GVHD. For instance, therapies aimed at modulating Treg differentiation or function could enhance GvL without exacerbating GVHD.

Neoantigen presentation is also a recognized factor in host immune response to cancer. Improved neoantigen presentation by class-II HLA alleles post-transplant was associated with improved OS in our CIBMTR cohort, despite only a minority of patients having mismatched class-II HLA alleles. Other studies have noted benefit from CD4 T cell responses independent of CD8 activities directed against neoantigens.^74^^,^^75^ This could potentially suggest that class-II allele mismatches leading to superior neoantigen presentation may lead to more durable benefit from bone marrow transplant and may be less prone to inducing CD8-driven cytotoxic autoimmunity. However, some class-II HLA alleles have been associated with humoral autoimmune diseases.^76^^,^^77^^,^^78^ Further evaluation is needed to understand the potential benefits and risks of selecting donor HLA alleles to more optimally target neoantigens in MDS.

Notably, in contrast to the survival benefit of improved neoantigen presentation by class-II HLA alleles post-transplant, none of our PHBR-based measures of overall immunogenicity were significantly predictive of relapse-free survival or OS in an initial univariate analysis The latter observation is consistent with the idea that established tumors evolve to escape adaptive immunity after a duration of homeostasis wherein immunogenic neoantigens become either pruned or tolerated by the immune system.^79^ Therefore, the introduction of novel HLA alleles that modify which neoantigens are immunogenic following allo-HSCT presents a novel immune challenge to a tumor. Although clinical trials of immune checkpoint inhibitors in higher-risk MDS have been largely disappointing, lessons from other cancers where these therapies are successful suggest that the immunogenicity of driver mutations is a more critical determinant of response than that of passengers.^24^^,^^26^ This principle underscores the need for a wider analysis of mutation immunogenicity, such as through PHBR scores, to better understand and predict responses to other immunotherapies in MDS, including allo-HSCT.

In conclusion, our study reveals a novel and robust association between autoimmune-associated class-I HLA alleles and longer relapse-free survival, as well as between a change in neo-antigen presentation post-transplant and longer OS after allo-SCT for MDS. These findings hold potential for new HLA matching strategies and therapeutic approaches, improving outcomes for patients undergoing transplantation.

### Limitations of the study

There are several limitations to our study. First, because our analysis was limited to a panel of common driver mutations, which does not reflect the complete immunogenicity of a patient’s mutanome, nor key biomarkers of patient risk, further investigations of a broader spectrum of molecular alterations is necessary in the future. Additionally, the frequency of HLA-mismatched donors was low, limiting the statistical power of analyses related to improved PHBR-II scores. These limitations also prevent us from calculating the clinically significant IPSS-M score^27^ and including it as a covariate in our analyses. Furthermore, the sample size for some sub-analyses related to these molecular features was limited, which necessitates caution against the overinterpretation of these specific findings pending validation in larger cohorts. Additional analysis of HLA effects in the context of the IPSS-M scores should also be the subject of future work investigating how these measures may complement each other for advanced patient risk stratification. Second, the study was limited to MDS patients, and it would be of interest to determine if these results, i.e., autoimmune alleles and change in neoantigen presentation post-transplant, are important predictors of outcome of patients with AML or with lymphoid/other malignancies after transplant. Finally, this study was conducted only in Caucasian patients. It is well-established that the frequencies of HLA alleles vary significantly across different ancestral populations. Therefore, the specific associations we observed may not be generalizable to other populations. Additional analysis is needed to replicate these results in a more ethnically diverse cohort.

## Resource availability

### Lead contact

Requests for further information and resources should be directed to and will be fulfilled by the lead contact, Hannah Carter (hkcarter@ucsd.edu).

### Materials availability

This study did not generate new unique reagents.

### Data and code availability

• The CIBMTR only releases deidentified datasets that comply with all relevant global regulations regarding privacy and confidentiality.
• Code to reproduce models, analyses, and figures can be found at the following Github repository: https://github.com/cartercompbio/CIBTMR_AutoImmuneAnalysis, https://doi.org/10.5281/zenodo.17428751.
• This paper does not report any additional resources.

## Acknowledgments

This work was supported by 10.13039/100014599Mark Foundation for Cancer Research grant 18-022-ELA to H.C. R.K. is funded in part by 5U01CA180888-08 and 5UG1CA233198-05. J.D. is supported by K01 HL164972. CIBMTR is supported primarily by the 10.13039/100007197Public Health Service U24CA076518 from the 10.13039/100000054National Cancer Institute (NCI), the 10.13039/100000050National Heart, Lung and Blood Institute (NHLBI), and the 10.13039/100000060National Institute of Allergy and Infectious Diseases (NIAID); 75R60222C00011 from the 10.13039/100000102Health Resources and Services Administration (HRSA); and N00014-23-1-2057 and N00014-24-1-2057 from the 10.13039/100000006Office of Naval Research. Support is also provided by the 10.13039/100008980Medical College of Wisconsin, 10.13039/100026309NMDP, 10.13039/100001634Gateway for Cancer Research, 10.13039/100018758Pediatric Transplantation and Cellular Therapy Consortium, and from the following commercial entities: AbbVie; Actinium Pharmaceuticals, Inc.; Adaptive Biotechnologies Corporation; ADC Therapeutics; Adienne SA; Alexion; AlloVir, Inc.; Amgen, Inc.; Astellas Pharma US; AstraZeneca; Atara Biotherapeutics; BeiGene; BioLineRX; Blue Spark Technologies; bluebird bio, Inc.; Blueprint Medicines; Bristol Myers Squibb Co.; CareDx, Inc.; CSL Behring; CytoSen Therapeutics, Inc.; DKMS; Elevance Health; Eurofins Viracor, DBA Eurofins Transplant Diagnostics; Gamida-Cell, Ltd.; Gift of Life Biologics; Gift of Life Marrow Registry; GlaxoSmithKline; HistoGenetics; Incyte Corporation; Iovance; Janssen Research & Development, LLC; Janssen/Johnson & Johnson; Jasper Therapeutics; Jazz Pharmaceuticals, Inc.; Karius; Kashi Clinical Laboratories; Kiadis Pharma; Kite, a Gilead Company; Kyowa Kirin; Labcorp; Legend Biotech; Mallinckrodt Pharmaceuticals; Med Learning Group; Medac GmbH; Merck & Co.; Mesoblast; Millennium, the Takeda Oncology Co.; Miller Pharmacal Group, Inc.; Miltenyi Biotec, Inc.; MorphoSys; MSA-EDITLife; Neovii Pharmaceuticals AG; Novartis Pharmaceuticals Corporation; Omeros Corporation; OptumHealth; Orca Biosystems, Inc.; OriGen BioMedical; Ossium Health, Inc.; Pfizer, Inc.; Pharmacyclics, LLC, An AbbVie Company; PPD Development, LP; REGiMMUNE; Registry Partners; Rigel Pharmaceuticals; Sanofi; Sarah Cannon; Seagen Inc.; Sobi, Inc.; Stemcell Technologies; Stemline Technologies; STEMSOFT; Takeda Pharmaceuticals; Talaris Therapeutics; Vertex Pharmaceuticals; Vor Biopharma Inc.; Xenikos BV.

## Author contributions

Concept and study design: data processing, T.S., T.Z., J.D., Y.-T.B., Z.C., P.A., W.S.; analysis and machine learning, T.S., R.K., T.Z., S.R.S., A.M.G., W.S., H.C.; supervision, R.K., W.S., H.C.; manuscript writing, all authors; scientific and editorial feedback, R.K., S.S., W.S., H.C.

## Declaration of interests

Dr. Kurzrock has received research funding from Boehringer Ingelheim, Debiopharm, Foundation Medicine, Genentech, Grifols, Guardant, Incyte, Konica Minolta, Medimmune, Merck Serono, Omniseq, Pfizer, Sequenom, Takeda, and TopAlliance and from the NCI; as well as consultant and/or speaker fees and/or advisory board/consultant for Actuate Therapeutics, AstraZeneca, Bicara Therapeutics, Inc., Biological Dynamics, Caris, Datar Cancer Genetics, Daiichi, EISAI, EOM Pharmaceuticals, Iylon, LabCorp, Merck, NeoGenomics, Neomed, Pfizer, Precirix, Prosperdtx, Regeneron, Roche, TD2/Volastra, Turning Point Therapeutics, X-Biotech; has an equity interest in CureMatch Inc.; serves on the Board of CureMatch and CureMetrix, and is a co-founder of CureMatch.

## STAR★Methods

### Key resources table

**Table:** 

REAGENT or RESOURCE	SOURCE	IDENTIFIER
**Software and algorithms**		

Reproducibility code for manuscript	This paper	https://doi.org/10.5281/zenodo.17428751
R Programming Language v4.4	R Foundation for Statistical Computing	https://www.R-project.org/
dplyr 1.1.4	CRAN	https://CRAN.R-project.org/package=dplyr
survminer 0.4.9	CRAN	https://CRAN.R-project.org/package=survminer
survival 3.5-8	CRAN	https://CRAN.R-project.org/package=survival
tidyverse 2.0.0	CRAN	https://CRAN.R-project.org/package=tidyverse
ggpubr 0.6.0	CRAN	https://CRAN.R-project.org/package=ggpubr
rcmdr 2.9-2	CRAN	https://CRAN.R-project.org/package=rcmdr
readxl 1.4.3	CRAN	https://CRAN.R-project.org/package=readxl
ggfortify 0.4.17	CRAN	https://CRAN.R-project.org/package=ggfortify
pheatmap 1.0.12	CRAN	https://CRAN.R-project.org/package=pheatmap
ggpmisc 0.5.6	CRAN	https://CRAN.R-project.org/package=ggpmisc
dplyr 1.1.4	CRAN	https://CRAN.R-project.org/package=dplyr
survminer 0.4.9	CRAN	https://CRAN.R-project.org/package=survminer
survival 3.5-8	CRAN	https://CRAN.R-project.org/package=survival
tidyverse 2.0.0	CRAN	https://CRAN.R-project.org/package=tidyverse
ggpubr 0.6.0	CRAN	https://CRAN.R-project.org/package=ggpubr
rcmdr 2.9-2	CRAN	https://CRAN.R-project.org/package=rcmdr
readxl 1.4.3	CRAN	https://CRAN.R-project.org/package=readxl
ggfortify 0.4.17	CRAN	https://CRAN.R-project.org/package=ggfortify
pheatmap 1.0.12	CRAN	https://CRAN.R-project.org/package=pheatmap
ggpmisc 0.5.6	CRAN	https://CRAN.R-project.org/package=ggpmisc

### Method details

#### Data sources

The Center for International Blood & Marrow Transplant Research (CIBMTR) is a research affiliation between the Medical College of Wisconsin and NMDP. It facilitates critical research through medical, scientific, and statistical expertise and represents a network of more than 330 participating centers, a database with clinical data on 575,000 patients, and a biospecimen repository. The CIBMTR database yielded a cohort of 494 patients with HLA typing and driver gene somatic sequencing information. Studies conducted by the CIBMTR are performed in compliance with all applicable federal regulations pertaining to the protection of human research participants.

#### Data availability statement

The CIBMTR supports accessibility of research in accord with the National Institutes of Health Data Sharing Policy and the National Cancer Institute Cancer Moonshot Public Access and Data Sharing Policy. The CIBMTR only releases deidentified data sets that comply with all relevant global regulations regarding privacy and confidentiality.

#### Study design

A cohort of 494 patients was assembled from patients who reported to the CIBMTR with banked pre-alloHCT whole blood samples between 2014-2018. Both donor and recipient had available high resolution HLA typing available and mutation panel sequencing performed using whole genome sequencing. The objective of this large retrospective study was to investigate modifiers of cGVHD, specifically via changes in mutation presentation and the presence or absence of autoimmune HLA alleles. Eligible patients included graft recipients from donors with at least 6/8 HLA alleles matched, where loci considered included HLA-A, HLA-B, HLA-C, and HLA-DRB1. Donors included HLA-matched siblings, HLA-matched unrelated donors, HLA-mismatched siblings, and HLA mismatched unrelated donors. A validation cohort (termed the UCSD cohort) was constructed by reanalyzing data from 59 patients with available Foundation Medicine (Cambridge, MA) panel somatic mutation calling and HLA typing that received a bone marrow transplant as part of their standard of care at the University of California, San Diego, under the PREDICT trial (ClinicalTrials.gov Identifier: NCT02478931). This reanalysis was performed in accordance with UCSD institutional review board guidelines.

#### Somatic sequencing and HLA genotyping

Patients had their HLA genotyped via OCTOPUS (https://doi.org/10.1038/s41587-021-00861-3)^80^ as part of their sequencing schema. Detailed methods for sample preparation and sequencing can be found in *Zhang* et al. 2023.^81^ Briefly, all samples were collected in ACD-A tubes before the administration of the preparative conditioning regimen prior to transplantation and shipped overnight at ambient temperature to the CIBMTR Research Repository, aliquoted on the day of receipt and stored frozen at -80C or in N2(l). DNAs were extracted using either the Qiagen Puregene Method or DNA Blood Kit on the Perkin Elmer Chemagic 360. Qiagen Puregene kit was used for DNA extraction with a DNA yield of 100-500ng/ul DNA per sample. Whole genome sequencing was conducted by Broad Institute using a modified version of the Illumina TruSeq PCR-Free LT Library Prep method (Illumina), however the present study was limited to a panel of 508 MDS driver genes analogous to the FoundationOne Heme gene panel.^82^

#### Patient and treatment characteristics

Patients were included if they had a diagnosis of MDS and had sequencing data. Major clinical factors evaluated in this analysis included age, donor age, sex, donor sex, disease status at time of transplant as defined by IPSS-R (very low, low, intermediate, high, very high), donor type, conditioning regimen intensity (myeloablative, reduced intensity, non-myeloablative), acute graft-versus-host disease as defined by Przepiorka et al.,^83^ and chronic graft-versus-host disease. Chronic GVHD severity (mild, moderate, or severe) was classified according to CIBMTR definitions, reflecting the maximum severity documented over longitudinal follow-up, as determined as best clinical judgment. Additional clinical variables included stem cell source (bone marrow or peripheral blood), and ethnicity. Primary endpoints considered were relapse-free survival and overall survival.

### Quantification and statistical analysis

#### Auto-immune allele curation

An initial list of HLA alleles with an association to autoimmune disease were selected from Castro et al.^4^ We then refined this set using the following criteria: first, only alleles associated with an increased risk of autoimmune disease were included, consistent with evidence that autoimmune alleles increasing disease risk are better at presenting self antigens.^84^ Second, we determined a frequency threshold with which to selectively retain autoimmune alleles, because low frequency alleles (selected against in the general population) will necessarily have stronger autoimmune effects.^85^ Since there is no commonly accepted threshold used for distinguishing functionally significant autoimmune alleles based on population frequency, we employed an unbiased statistical approach to objectively determine this cutoff. We determined an optimal threshold ***t*** using the following formula from *McDonald & Kreitman* (Nature 1991)^86^ and implemented the following protocol outlined in Fijarczyk et al. (Mol. Ecol. 2015)^87^ where O_i_ is the observed distribution of an allele, E_i_ is the expected distribution (AF=0.5), k is the number of cells in the contingency table, and N is the total number of observations.

1) First calculate the G-test statistic
  (Equation 1) $$G=2\sum O_{i}\;\ln\left(\frac{O_{i}}{E_{i}}\right)$$
2) Then use the Williams correction to reduce bias in smaller sample sizes
  (Equation 2) $$G_{corrected}=\frac{G}{1+\frac{k^{2}-1}{6N}}$$

This resulted in an AF threshold ***t***=0.3078 where P<0.05 where autoimmune alleles with an AF larger than ***t*** were dropped. Remaining alleles with no observed presence in the CIBMTR cohort were also necessarily dropped. Finally, we removed alleles solely associated with rheumatoid arthritis and type 1 diabetes due to their ubiquity. This resulted in a list of 14 HLA autoimmune alleles: 9 class-I and 5 class-II autoimmune alleles.

#### Mutation affinity analysis

HLA types and somatic driver mutations for 494 patients were obtained from CIBMTR. Nonsynonymous single nucleotide variations, indels, and frameshift mutations were considered for mutation affinity analysis. These mutations were computationally assessed for their predicted binding affinity using NetMHCpan 4.1 and NetMHCIIpan 4.0^88^ for patient and donor HLA types. These binding affinities were then used to construct PHBR scores described previously^23^^,^^25^ for the patient’s set of mutations using both donor and recipient HLA alleles (with PHBR representing a the MHC ability to present cancer mutations to CD8^+^ T cells (with lower PHBR indicating better presentation)). Patients who did not have a perfect 8/8 HLA match for their allo-HSCT had measurable differences in their PHBR scores which could be investigated for associations with outcome. Changes in PHBR presentation scores were calculated for 44 (9%) patients that had a difference between HLA donor/host alleles and final change in PHBR scores were reported as the mean change in score across all mutations per patient (Donor PHBR - Recipient PHBR), such that each patient had a continuous score reflecting the total change in presentation of their mutations by MHC-I and MHC-II respectively. We then took the inverse of these scores and termed them “Improved PHBR-I” and “Improved PHBR-II” such that a positive score would reflect an improvement in presentation and a negative would reflect a degradation in presentation. This term “Improved PHBR” conveys that an increase in the metric indicates an improvement in antigen presentation by the donor MHC. This stands in contrast to the raw PHBR score where a lower value indicates more effective presentation.

#### Survival analyses

Kaplan-Meier curves were used to compare outcome in non-time-dependent clinical variables such as auto-immune present/absent groups, and the log-rank test was used to generate P-values. Our multivariable Cox proportional hazard (CoxPH) analysis was conducted using the methodology previously described^89^: in separate analyses for both relapse-free survival and overall survival, all clinical and experimental covariates were subjected to CoxPH analysis and the covariate with the highest P-value (least significant) was iteratively dropped until the Akaike information criterion was maximized. This allows us to obtain the model that best fits the data with the minimal parameters. To prevent a spurious time-dependent bias for acute and chronic graft versus host disease, these variables were encoded with their time-to-event information using the “tmerge” function.^90^ Using the resulting relapse-free survival CoxPH model, clinical and experimental feature interaction P-values were calculated using the same time dependent CoxPH test without covariates in a pairwise fashion before Bonferroni multiple testing correction was applied. Due to the inherent immortal time bias involved in analyzing the interaction between cGVHD and Class-I autoimmune alleles, we used Simon-Makuch^71^ plots rather than Kaplan-Meier curves for visualization purposes, and Mantel-Byar^72^ statistical tests to calculate the hazard associated with each group. Pooled meta-analyses for comparable results between the CIBMTR cohort and UCSD cohort were performed by ingesting hazard ratios, and standard errors into R package rma.
