# Supplementary material for: Autoimmune HLA alleles and neoantigens predict myelodysplastic syndrome outcomes after allogeneic HSCT: A CIBMTR analysis
Source: iScience. 2025 Dec 18;29(1):114326. doi: 10.1016/j.isci.2025.114326 (PMC12804621; doi:10.1016/j.isci.2025.114326)
Supplement: Document S1. Figures S1–S14 and Tables S1 and S2 [file mmc1.pdf]

iScience, Volume *29*

Supplemental information

Autoimmune HLA alleles and neoantigens predict myelodysplastic syndrome outcomes after allogeneic HSCT: A CIBMTR analysis

Timothy Sears, Razelle Kurzrock, Tao Zhang, Jing Dong, Stephen R. Spellman, Aaron M. Goodman, Yung-Tsi Bolon, Zhongyuan Chen, Paul Auer, Wael Saber, and Hannah Carter

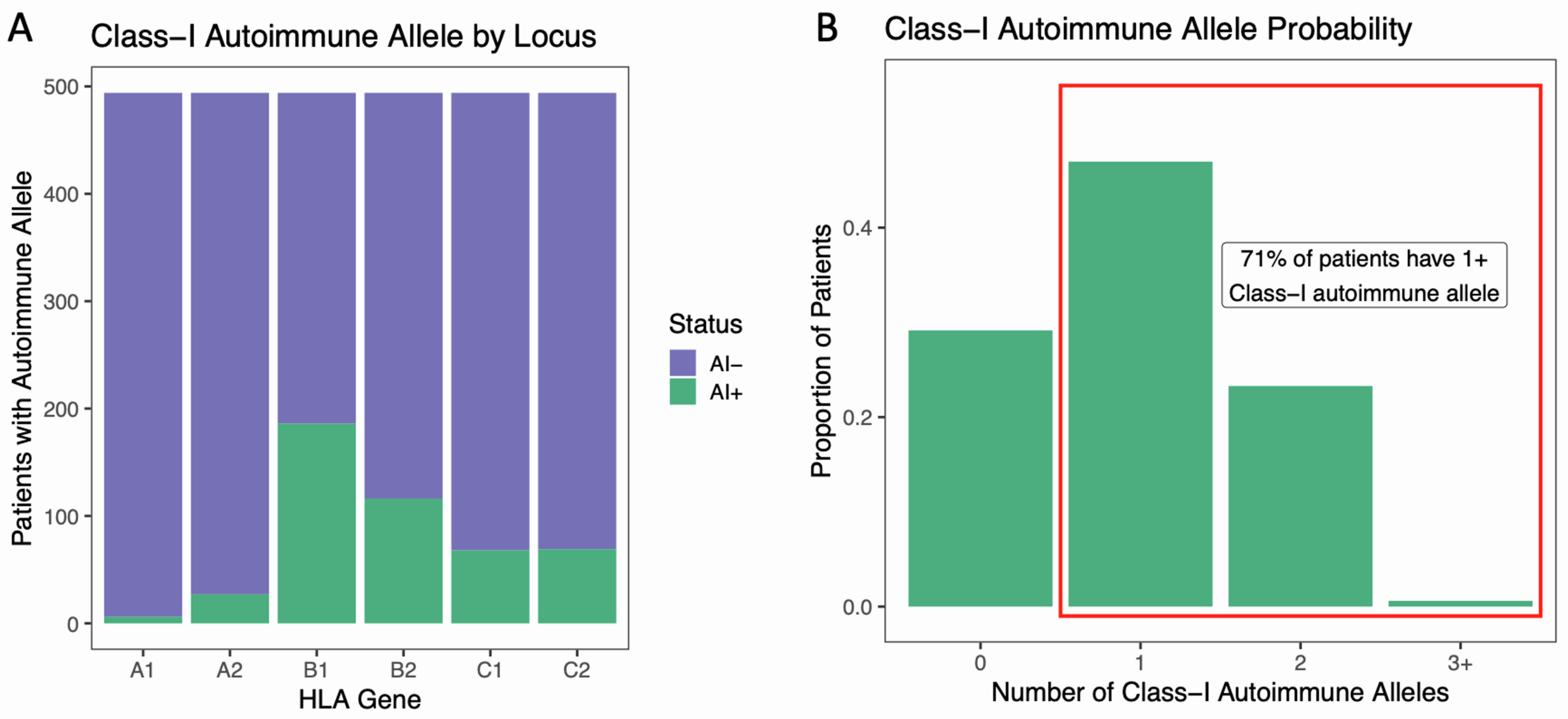

Fig S1: 6 total HLA loci enable a majority of patients to have at least 1 Class-I autoimmune allele. A) Number of patients with a Class-I autoimmune allele by specific HLA loci. **B)** Proportion of patients with at least one autoimmune allele is higher than any individual allele frequency.

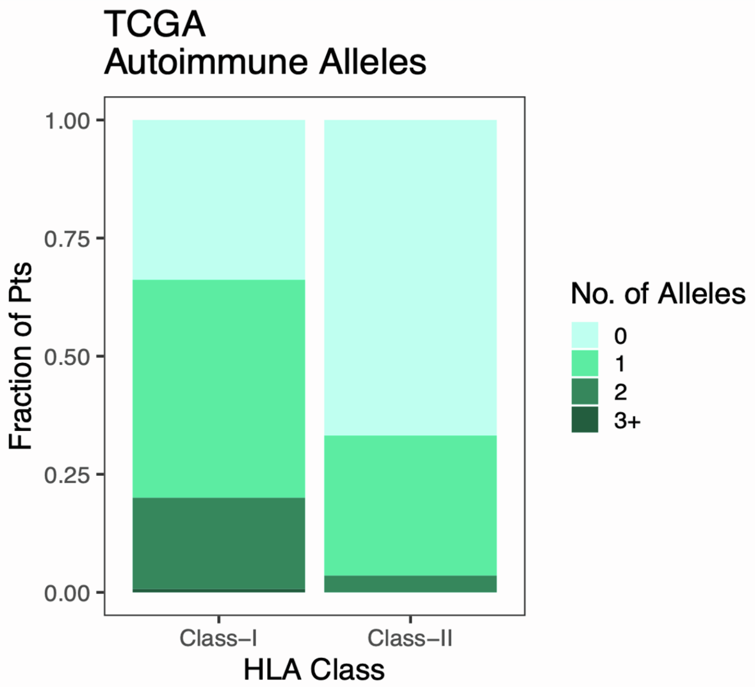

Fig S2: Frequency of HLA autoimmune alleles in TCGA LAML cohort

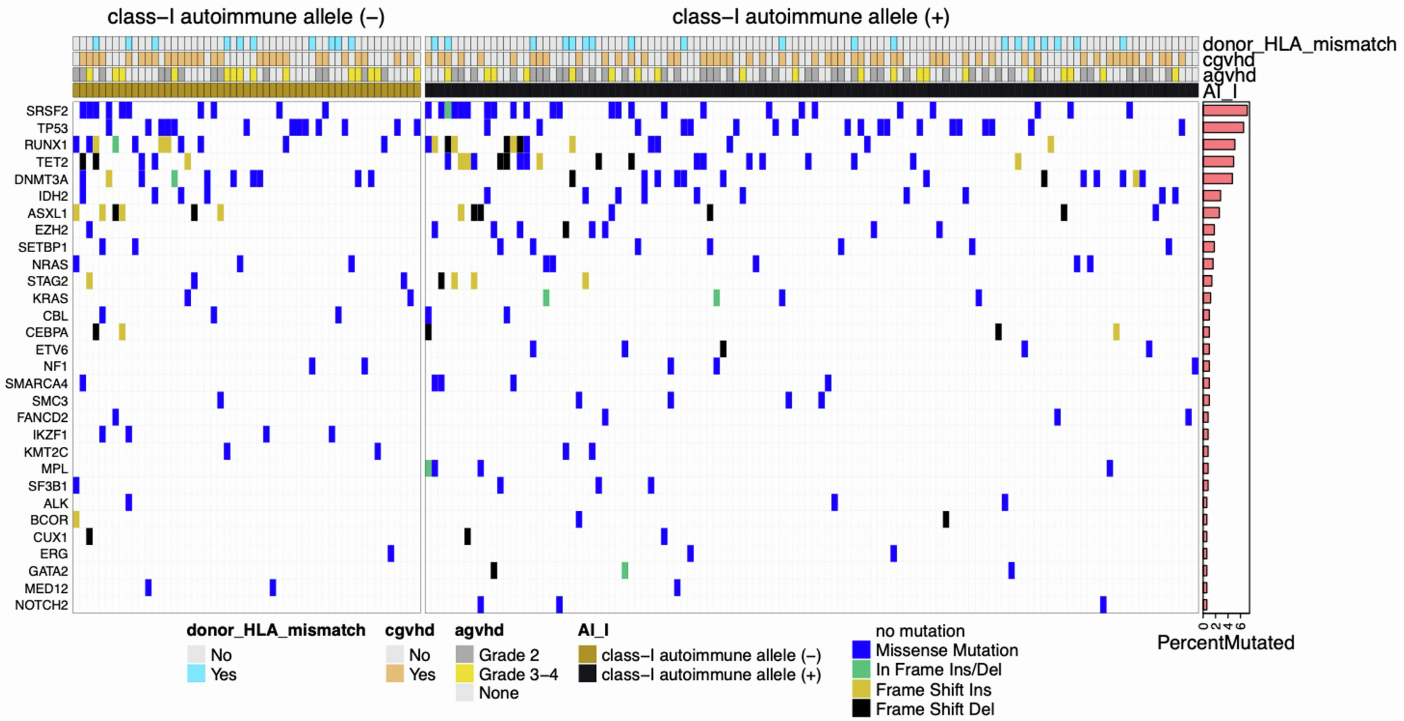

Fig S3: Oncoprint with top mutations from panel sequencing of CIBMTR cohort split by presence/absence of class-I autoimmune allele

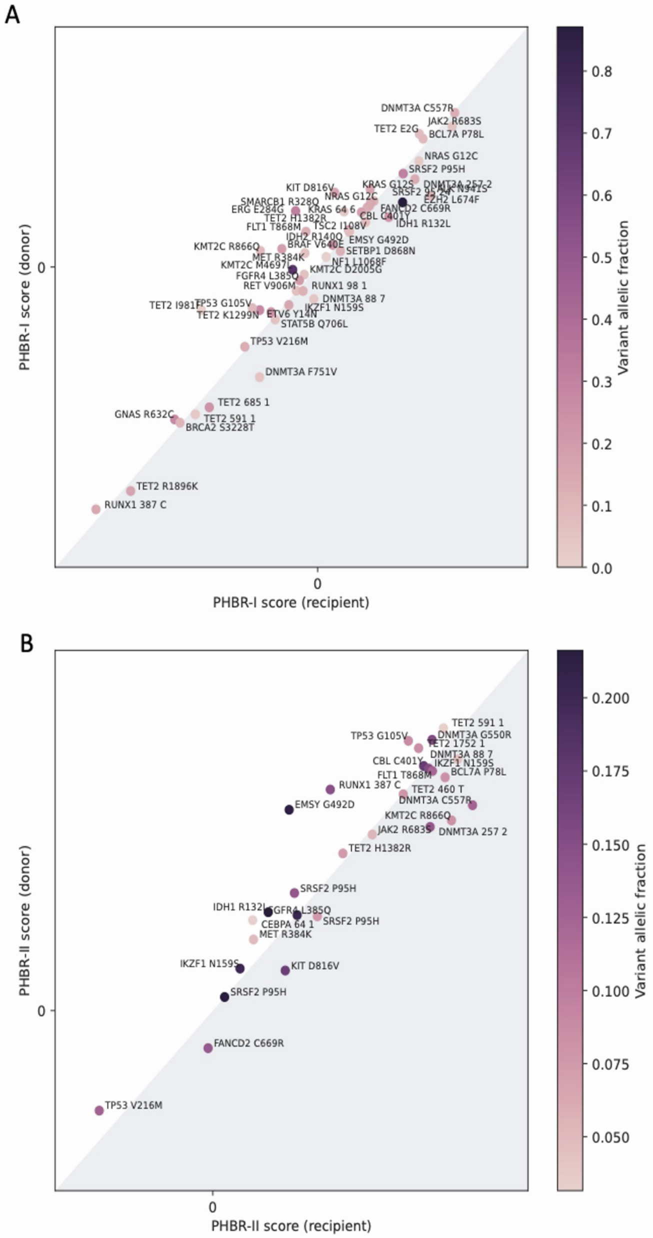

Fig S4: Changes in Patient Harmonic mean Best Rank (PHBR) scores for transplant recipients with donor HLA mismatch. A) Class-I HLA mismatch patients **B)** Class-II HLA mismatch patients

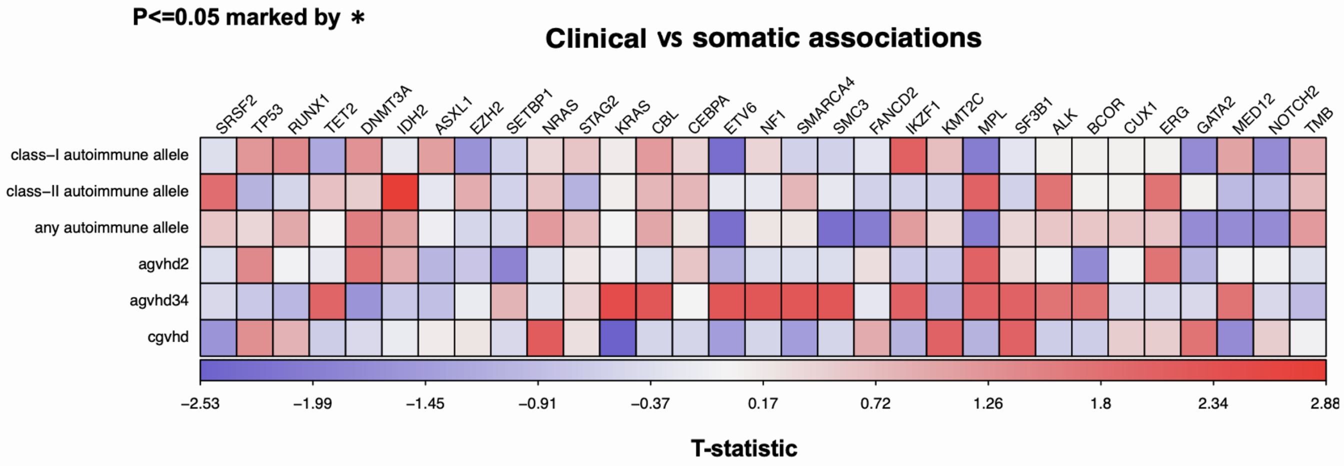

Fig S5: Association of somatic mutations with clinical features reveal no significant relationships between somatic landscape and autoimmunity.

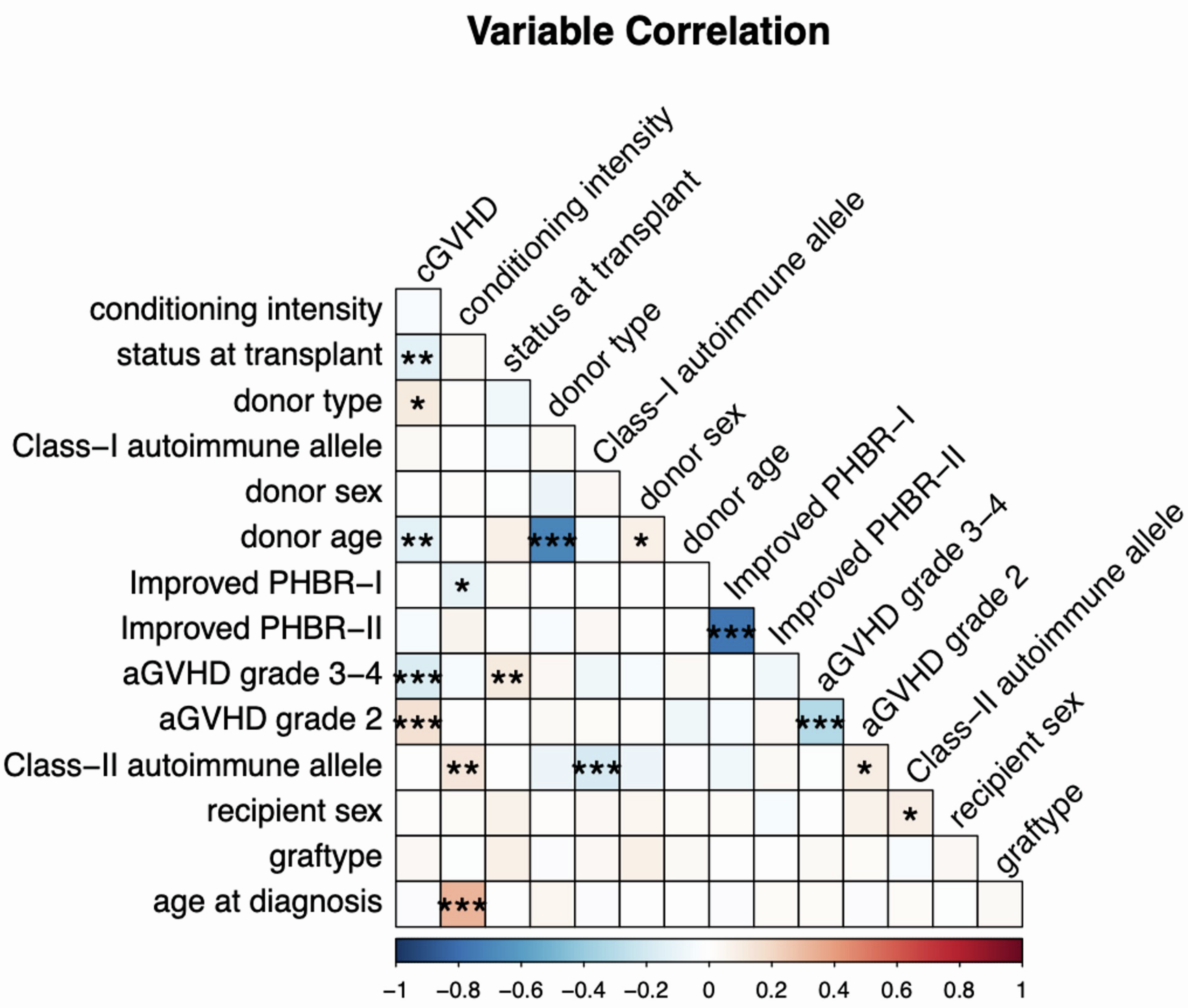

Fig S6: Correlation of clinical features reveals no relationship between autoimmune alleles and development of chronic graft vs. host disease (cGVHD)

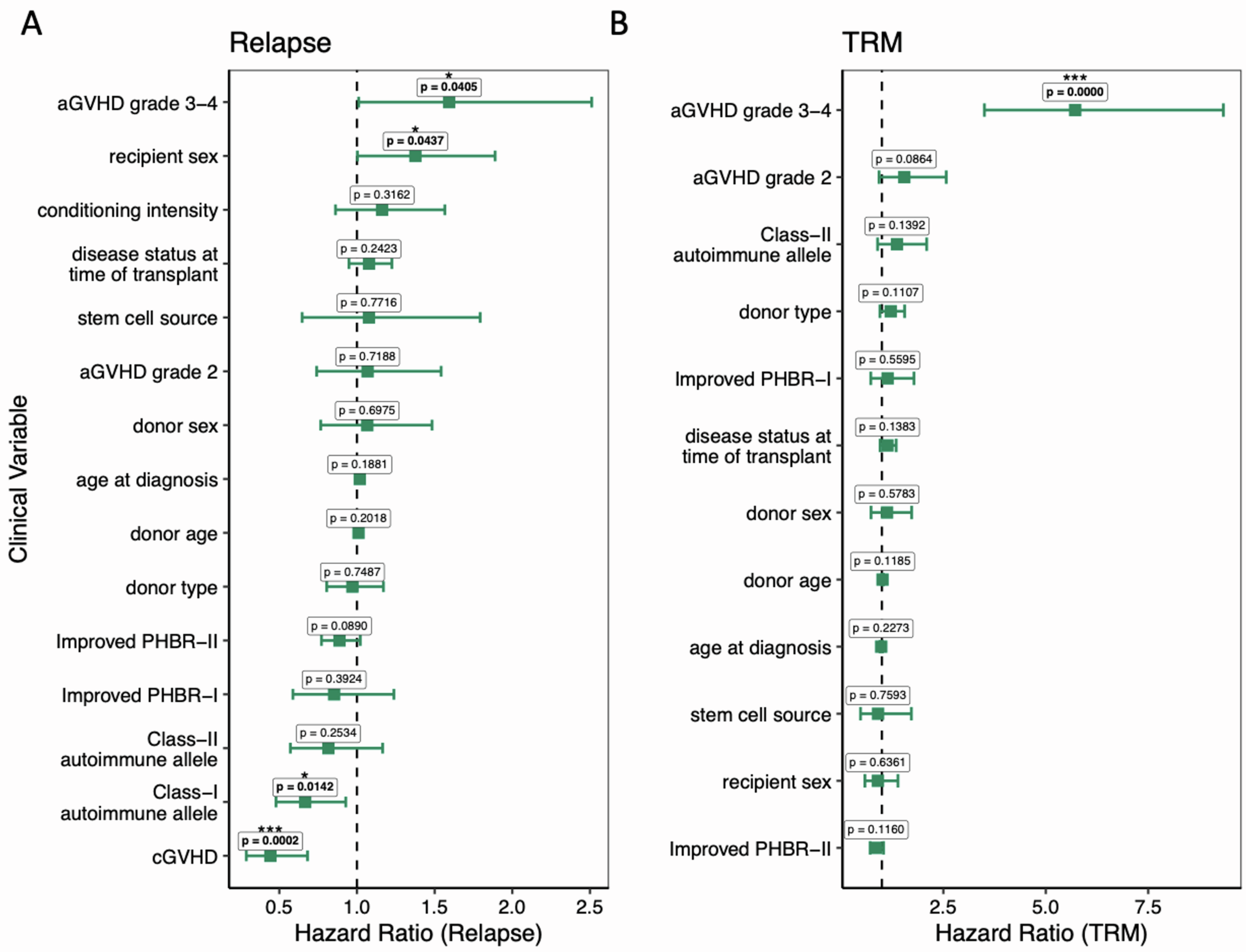

Fig S7. Time-dependent multivariable analysis of clinical variables on treatment related mortality (TRM) and relapse. A) Time-dependent multivariable analysis of clinical variables passing backwards feature selection with relationship to treatment related mortality. **B)** Time-dependent multivariable analysis of clinical variables passing backwards feature selection with relationship to overall relapse.

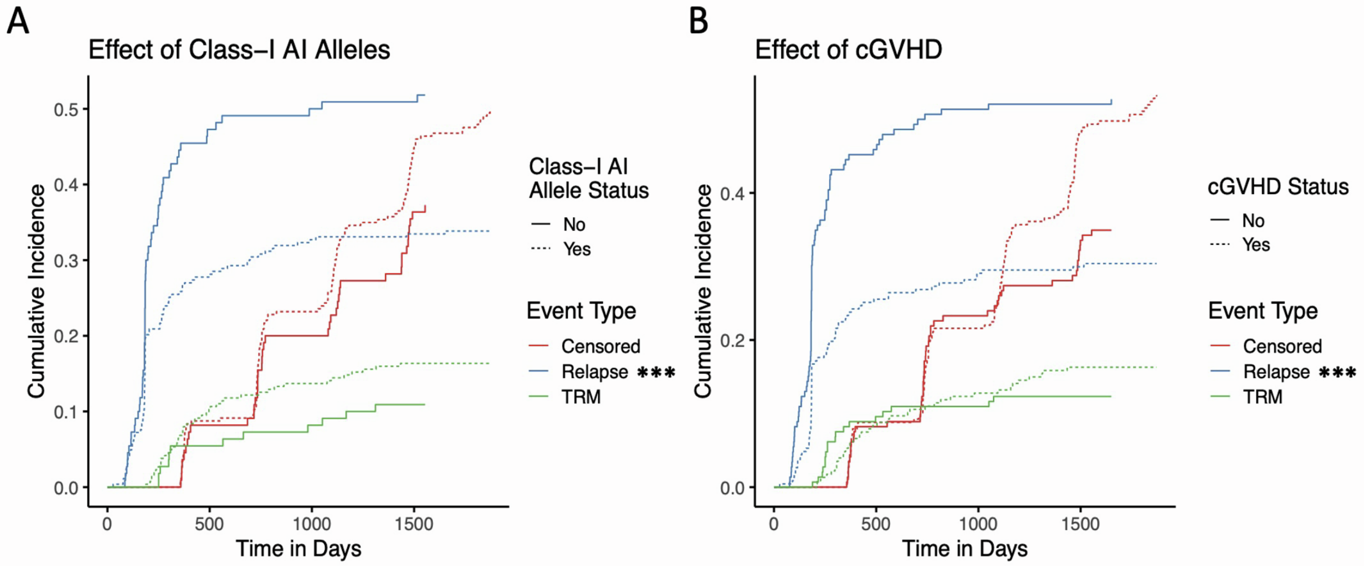

Fig S8: Individual Effects of Class-I AI Alleles and Chronic GVHD on Competing Risks. Cumulative incidence functions (CIFs) for Relapse and Transplant-Related Mortality (TRM) are shown stratified separately by **(A)** Class-I AI Allele status and **(B)** chronic Graft-versus-Host Disease (cGVHD) status (right panel). An asterisk (*) in the legend indicates a significant difference ($p < 0.05$) in the cumulative incidence of an event between the positive and negative groups, as determined by Gray's test.

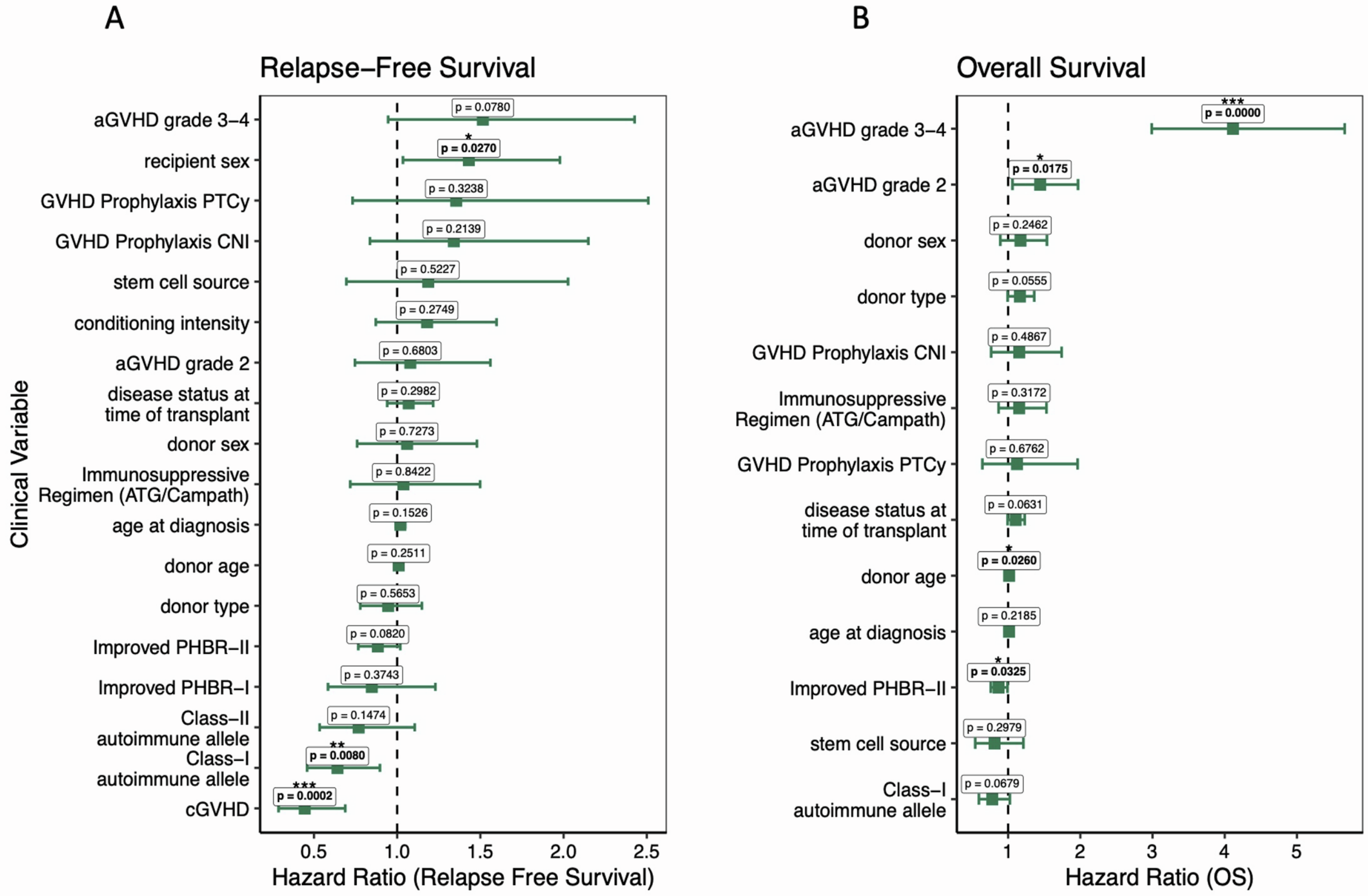

Fig S9: Multivariable analysis of relapse-free and overall survival with inclusion of immunomodulators. Time-dependent multivariable analyses demonstrate that the significant protective features of the primary model are maintained for both **(A) relapse-free survival** and **(B) overall survival** after including covariates for pre-transplant immunosuppression (**ATG/Campath**) and post-transplant GVHD prophylaxis (**PTCy**).

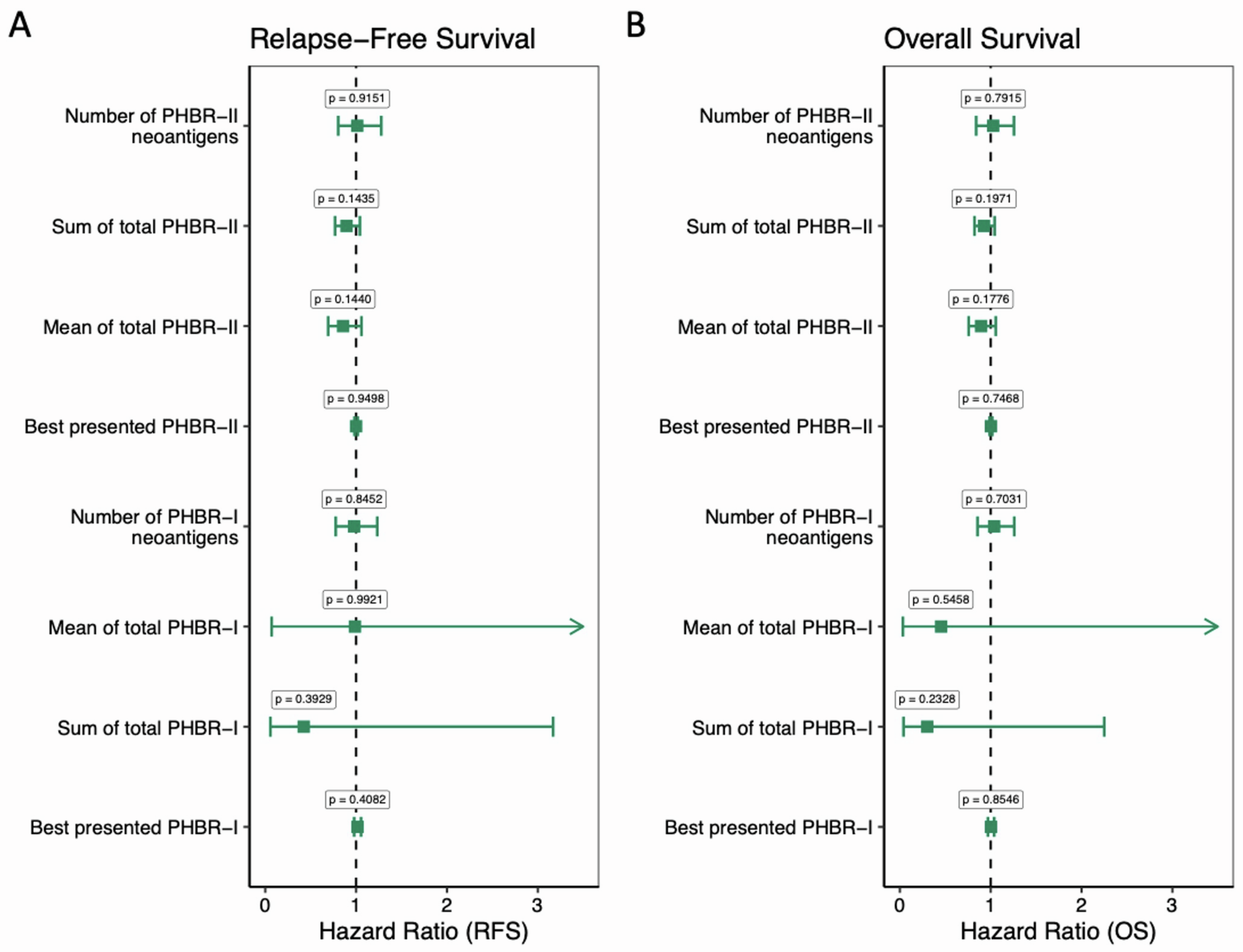

Fig S10: PHBR-based measures of overall immunogenicity fail to predict A) Relapse-free survival or **B)** Overall survival

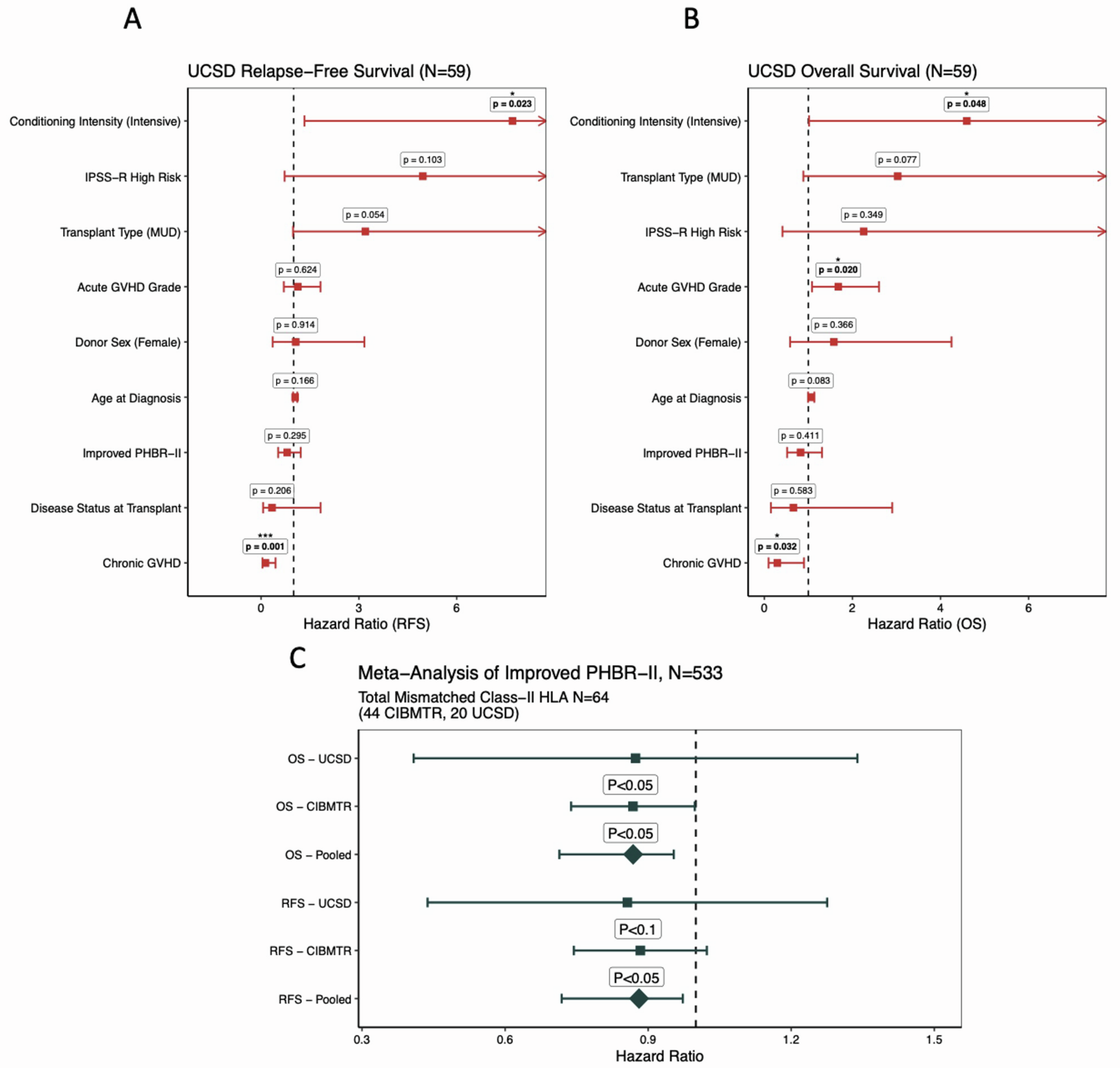

Fig S11: Validation of Improved PHBR-II in UCSD Cohort and Pooled Meta-Analysis with CIBMTR. **A)** Multivariable CoxPH analysis of clinical variables and improved PHBR-II in the UCSD cohort in Relapse-Free Survival. **B)** Multivariable CoxPH analysis of clinical variables and improved PHBR-II in the UCSD cohort in Overall Survival. **C)** Meta-Analysis of multivariable CoxPH results (pooled cohort)

A

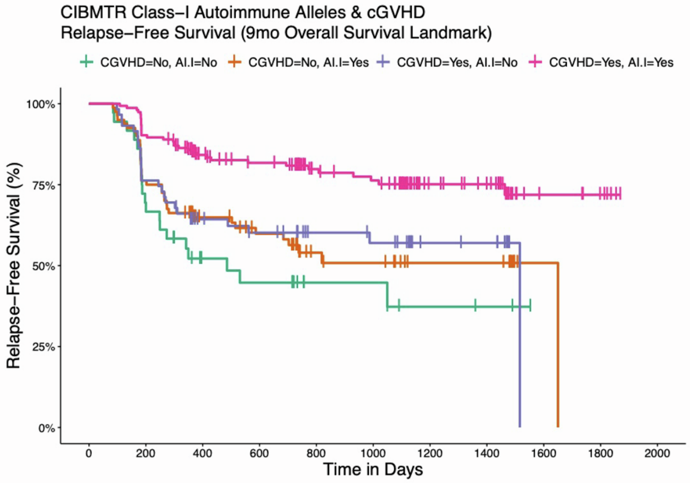

Number at Risk

	0	200	400	600	800	1000	1200	1400	1600	1800	2000
CGVHD=No, AI.I=No	36	25	14	12	6	6	3	2	0	0	0
CGVHD=No, AI.I=Yes	80	61	41	34	17	15	9	9	1	0	0
CGVHD=Yes, AI.I=No	59	45	32	28	20	18	10	9	0	0	0
CGVHD=Yes, AI.I=Yes	154	139	107	97	70	65	36	31	8	6	0

Time in Days

	AI.I=No, CGVHD=No	AI.I=No, CGVHD=Yes	AI.I=Yes, CGVHD=No
CGVHD=No, AI.I=Yes	0.227	NA	NA
CGVHD=Yes, AI.I=No	0.2023	0.793	NA
CGVHD=Yes, AI.I=Yes	<0.0001	<0.0001	<0.0001

B

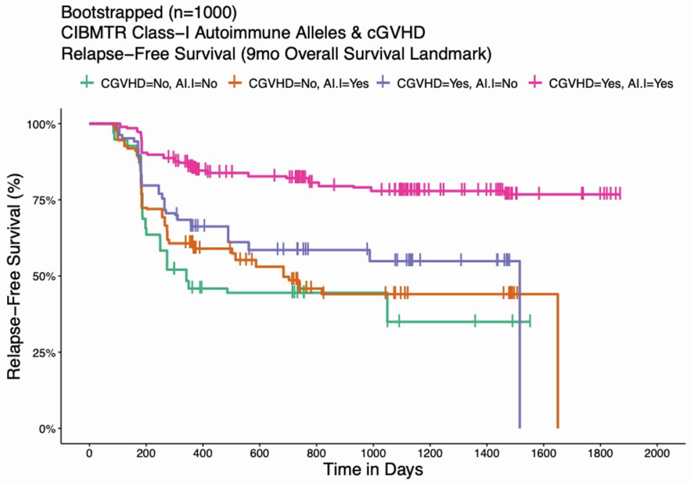

Number at Risk

	0	200	400	600	800	1000	1200	1400	1600	1800	2000
CGVHD=No, AI.I=No	96	63	33	32	14	14	8	7	0	0	0
CGVHD=No, AI.I=Yes	257	186	115	96	50	45	24	24	4	0	0
CGVHD=Yes, AI.I=No	187	149	106	90	65	60	27	24	0	0	0
CGVHD=Yes, AI.I=Yes	460	416	323	297	207	196	117	96	27	20	0

Time in Days

	AI.I=No, CGVHD=No	AI.I=No, CGVHD=Yes	AI.I=Yes, CGVHD=No
CGVHD=No, AI.I=Yes	0.2188	NA	NA
CGVHD=Yes, AI.I=No	0.0139	0.1081	NA
CGVHD=Yes, AI.I=Yes	<0.0001	<0.0001	<0.0001

Fig S12. 9 month landmark with and without bootstrap reveals synergistic protective effect of cGVHD and AI-I (+) against relapse. A) Kaplan-meier plot of four way comparison between presence or absence of cGVHD and class-I autoimmune allele (AI-I). Pvals computed with the log-rank test. **B)** Kaplan-meier plot of bootstrapped validation (n=1000) of four way comparison between presence or absence of cGVHD and AI-I allele. Pvals computed with the log-rank test.

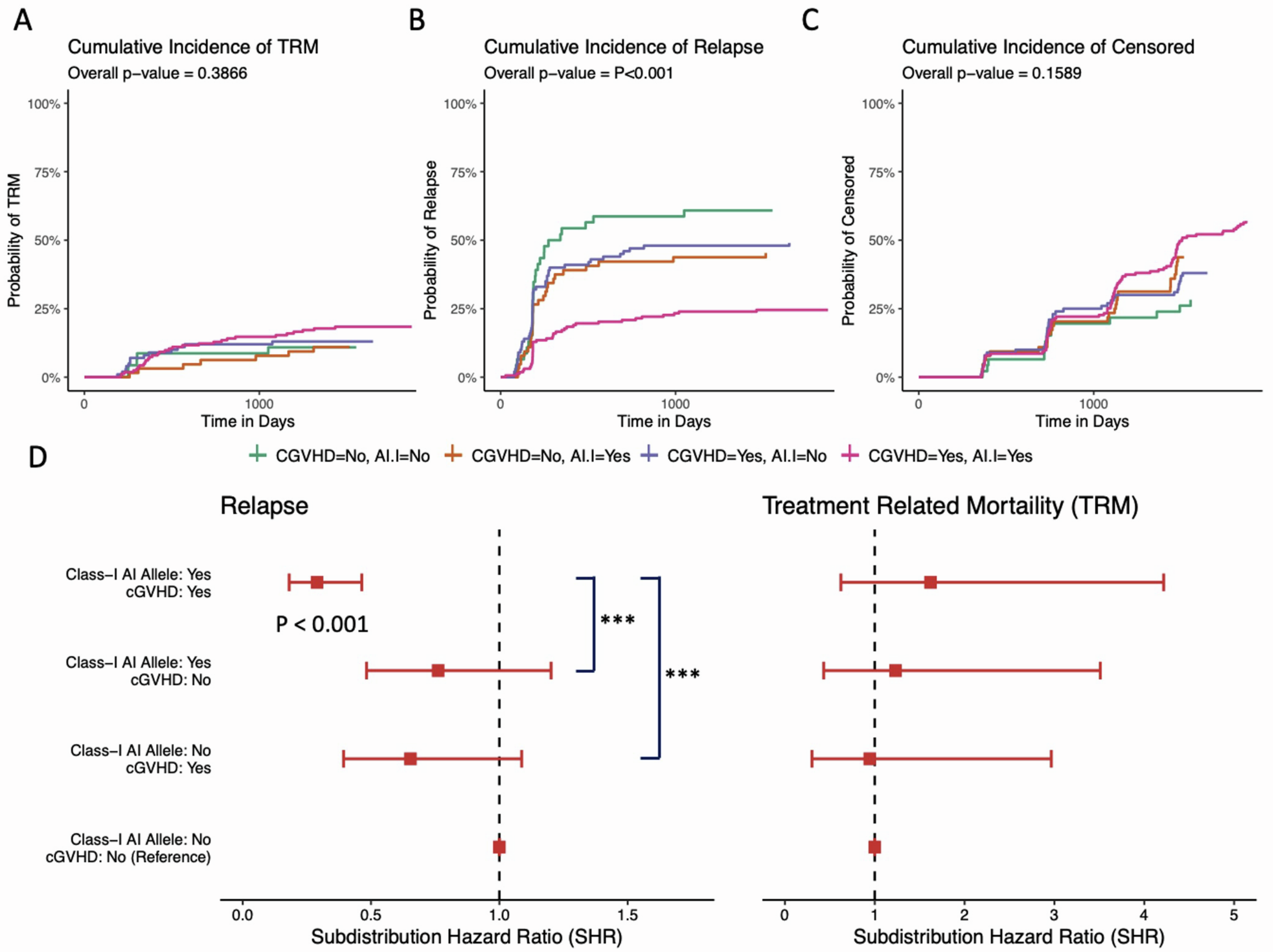

Fig S13: Interaction Between Class-I AI Alleles and Chronic GVHD on Competing Risk Outcomes. (A-C) Cumulative incidence functions (CIFs) for (A) Censored patients, (B) Relapse, and (C) Transplant-Related Mortality (TRM) are shown, stratified by the four interaction groups of Class-I AI Allele and cGVHD status. The overall p-value from Gray's test comparing the four curves is shown in the subtitle of each plot. **(D)** Forest plots display the Subdistribution Hazard Ratios (SHRs) and 95% confidence intervals from multivariable Fine-Gray competing risk models for Relapse (left panel) and TRM (right panel). The "Class-I AI Allele: No, cGVHD: No" group serves as the reference (SHR = 1.0). P-values shown are from pairwise comparisons of each group against the "Class-I AI Allele: Yes, cGVHD: Yes" group to assess the significance of the interaction.

A

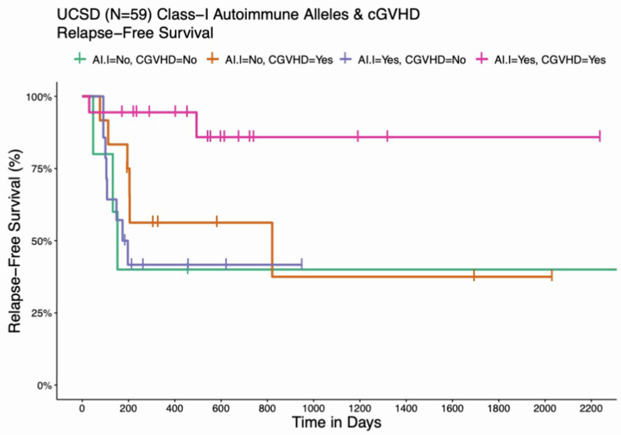

Number at Risk

	0	200	400	600	800	1000	1200	1400	1600	1800	2000	2200
AI.I=No, CGVHD=No	5	2	2	1	1	1	1	1	1	1	1	1
AI.I=No, CGVHD=Yes	12	8	4	3	3	2	2	2	2	1	1	0
AI.I=Yes, CGVHD=No	14	5	3	2	1	0	0	0	0	0	0	0
AI.I=Yes, CGVHD=Yes	18	16	13	7	3	3	2	1	1	1	1	1

Time in Days

	AI.I=No, CGVHD=No	AI.I=No, CGVHD=Yes	AI.I=Yes, CGVHD=No
AI.I=No, CGVHD=Yes	0.5139	NA	NA
AI.I=Yes, CGVHD=No	0.8837	0.3766	NA
AI.I=Yes, CGVHD=Yes	0.0111	0.0267	0.0033

B

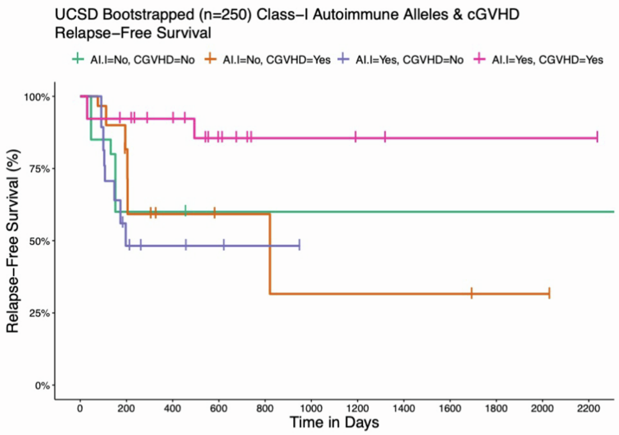

Number at Risk

	0	200	400	600	800	1000	1200	1400	1600	1800	2000	2200
AI.I=No, CGVHD=No	20	12	12	5	5	5	5	5	5	5	5	5
AI.I=No, CGVHD=Yes	60	40	22	15	15	8	8	8	8	5	5	0
AI.I=Yes, CGVHD=No	75	31	17	9	6	0	0	0	0	0	0	0
AI.I=Yes, CGVHD=Yes	90	74	62	33	17	17	12	7	7	7	7	7

Time in Days

	AI.I=No, CGVHD=No	AI.I=No, CGVHD=Yes	AI.I=Yes, CGVHD=No
AI.I=No, CGVHD=Yes	0.8051	NA	NA
AI.I=Yes, CGVHD=No	0.4671	0.0929	NA
AI.I=Yes, CGVHD=Yes	0.0013	<1e-04	<1e-04

Fig S14: Validation of Class-I Autoimmune Allele & cGVHD interaction visualized as Kaplan-Meier curve of relapse-free survival of A) UCSD validation cohort of MDS patients split into four categories by yes/no cGVHD and yes/no Class-I Autoimmune Allele presence. Log-rank pval results shown in table at bottom. **B)** The UCSD validation cohort bootstrapped up to 250 sampled with replacement.

Table S1

Characteristics	Autoimmune Allele (+)	Autoimmune Allele (-)	P-value (test)
**Patient age (year) - median (min-max)**	62.61 (17.62-73.65)	61.83 (41.75-78.94)	0.64 (t-test)
**Sex - no. (%)**			
Male	266 (63)	50 (68)	0.46 (chi-squared)
**MDS IPSS-R score pre transplant - no. (%)**			0.98 (chi-squared)
Very low	50 (12)	8 (11)	
Low	48 (11)	9 (12)	
Intermediate	105 (25)	18 (25)	
High	138 (33)	22 (30)	
Very high	61 (14)	13 (18)	
Missing	19 (5)	3 (4)	
**Time from diagnosis to HCT (month) - median (range)**	8.52 (0.63-175.92)	7.34 (2.53-263.06)	0.97 (chi-squared)
**Donor type - no. (%)**			0.28 (chi-squared)
HLA-identical sibling	56 (13)	9 (12)	
Other related	27 (6)	5 (7)	
Well-matched unrelated (8/8)	306 (73)	47 (64)	
Partially-matched unrelated (7/8)	28 (7)	11 (15)	
Mis-matched unrelated (≤6/8) or unknown	4 (1)	1 (1)	
**Stem cell source - no. (%)**			0.49 (chi-squared)
Bone marrow	48 (11)	11 (15)	
Peripheral blood	373 (89)	62 (85)	
**Regimen intensity - no. (%)**			0.25 (chi-squared)
Myeloablative	104 (25)	23 (32)	
Reduced intensity	263 (62)	45 (62)	
Non-myeloablative	39 (9)	5 (6)	
Missing	15 (4)	0 (0)	
**Median follow up of survivors (months) - median (range)**	33.85 (3.19-62.70)	35.69 (11.91-49.49)	0.64 (t-test, survivors only)
**Average number of panel mutations* (sd)**	1.34 (0.66)	1.27 (0.56)	0.39 (t-test)
**Median time (month) to graft versus host diseases (range)**			

Acute grade 2-4	1.12 (0.00-35.36)	1.18 (0.00-15.26)	0.94 (t-test)
Acute grade 3-4	1.15 (0.00-35.36)	1.02 (0.00-15.26)	0.075 (t-test)
Chronic	6.45 (0.00-41.32)	5.72 (0.00-17.67)	0.098 (t-test)

***Including frameshift mutations. Abbreviations: HMA=hypomethylating agents; HCT=hematopoietic stem cell transplantation; IPSS-R=international prognostic scoring system; KPS=Karnofsky performance status**

Table S2

CIBMTR Discovery Cohort			
**Characteristics**	**PHBR Change (N=44)**	**PHBR No Change (N=450)**	**P-value (test)**
**Patient age (year) - median (min-max)**	64 (43-73)	62 [18-79]	0.583 (t-test)
**Sex - no. (%)**			1.00 (chi-squared)
Male	28 (63.6%)	288 (64.0%)	
**MDS IPSS-R* score pre transplant - no. (%)**			0.758 (chi-squared)
Very low	6 (16.7%)	51 (12.8%)	
Low	8 (22.2%)	115 (28.7%)	
Intermediate	13 (36.1%)	147 (36.8%)	
High	6 (16.7%)	68 (17.0%)	
Very high	3 (8.3%)	19 (4.8%)	
**Time from diagnosis to HCT (month) - median (range)**	8.3 [0.6-94.9]	8.2 [1.6-263.1]	0.129
**Stem cell source - no. (%)**			<0.001 (chi-squared)
Bone marrow	14 (31.8%)	45 (10.0%)	
Peripheral blood	30 (68.2%)	405 (90.0%)	
**Regimen intensity - no. (%)**			<0.001 (chi-squared)
Myeloablative	7 (15.9%)	120 (26.7%)	
Reduced intensity	15 (34.1%)	293 (65.1%)	
Non-myeloablative	19 (43.2%)	25 (5.6%)	
Missing	3 (6.8%)	12 (2.7%)	
**Fraction with graft versus host diseases - no. (%)**			
Acute grade 2-4	21 (47.7%)	210 (46.7%)	0.931 (chi-squared)
Acute grade 3-4	12 (27.3%)	78 (17.3%)	0.135 (chi-squared)
Chronic	15 (34.1%)	223 (49.6%)	0.070 (chi-squared)

UCSD Validation Cohort			
**Characteristics**	**PHBR Change (N=18)**	**PHBR No Change (N=43)**	**P-value (test)**
**Patient age (year) - median (min-max)**	54.7 (22.1-73.6)	57.4 (28.5-75.2)	0.53 (t-test)

**Sex - no. (%)**			1.00 (chi-squared)
Male	11 (61.1%)	27 (62.8%)	
**MDS IPSS-R* score pre transplant - no. (%)**			0.929 (chi-squared)
Missing	10 (55.6%)	21 (48.8%)	
Low	1 (5.6%)	4 (9.3%)	
Intermediate	2 (11.1%)	6 (14.0%)	
High	1 (5.6%)	1 (2.3%)	
Very high	4 (22.2%)	11 (25.6%)	
**Time from diagnosis to HCT (month) - median (range)**	Not Available	Not Available	NA
**Stem cell source - no. (%)**			0.49 (chi-squared)
Bone marrow	4 (22.2%)	13 (30.2%)	
Peripheral blood	14 (77.8%)	28 (65.1%)	
**Regimen intensity - no. (%)**			1.00 (chi-squared)
Myeloablative	10 (55.6%)	23 (53.5%)	
Reduced intensity	8 (44.4%)	20 (46.5%)	
Non-myeloablative	0 (0%)	0 (0%)	
Missing	0 (0%)	0 (0%)	
**Median time (month) to graft versus host diseases (range)**			
Acute grade 2-4	6 (33.3%)	21 (48.8%)	0.407 (chi-squared)
Acute grade 3-4	4 (22.2%)	11 (25.6%)	1.00 (chi-squared)
Chronic	9 (50.0%)	29 (67.4%)	0.321 (chi-[illegible]

***IPSS-R=international prognostic scoring system**
